# The analysis of the prefrontal cortex and its facilitator role of violence: conclusions of a systematic review and meta-analysis based on neuroimaging results

**DOI:** 10.1007/s11682-026-01105-1

**Published:** 2026-03-10

**Authors:** Ángel Romero-Martínez, Carolina Sarrate-Costa, Luis Moya-Albiol

**Affiliations:** University of Valencia, Valencia, Spain

**Keywords:** Brain, Mental disorders, Personality disorders, Prefrontal cortex, Violence

## Abstract

The prefrontal cortex (PFC) is a key brain region for behavioral regulation; therefore, PFC abnormalities might partly be responsible for violence proneness. However, less is known about whether these alterations in PFC subregions are present in various types of individuals with a history of violent behaviors, including those who have committed violent crimes and those engaging in violent attacks against others, with or without mental or personality disorders. Therefore, the main objective of this review is to summarize the key findings from neuroimaging research that measures whether differences in the PFC exist between different samples of violent and criminal adults compared to non-violent individuals. It also examines the relationship between the PFC and violence-related concepts. A systematic review was conducted following the PRISMA quality criteria for reviews, using four digital databases and complemented with a snowballing search process. A total of 86 papers that met all the inclusion criteria were finally included. Most of these studies were conducted with men. Most individuals with histories of violent behavior (with and without mental and personality disorders) exhibited reduced volume in the orbitofrontal cortex compared to non-violent individuals. Other alterations included a lower synthesis of 5-HT in the OFC, along with higher levels of 5-HT2A receptors in the OFC and (dorsolateral PFC) DLPFC. However, these serotoninergic differences were only observed in a few groups of violent individuals, particularly those with high impulsivity. Violent individuals also exhibited different resting-state functional connectivity patterns between the OFC and lateral PFC (both dorsal and ventral) with the amygdala and cerebellum compared to non-violent individuals. This review specifies which PFC alterations may be generalizable and specific to different groups of violent individuals, with and without mental and personality disorders.

**Supplementary Information:**

The online version contains supplementary material available at 10.1007/s11682-026-01105-1.

## Introduction

The prefrontal cortex (PFC) constitutes the anterior region of the frontal lobe and makes up an extremely large portion of the brain’s cortex (Diamond, 2002), accounting for approximately 30% of the human cortex (Kolk & Rakic, 2022). It can be divided into three main subdomains based on its structural and functional differences: the medial PFC (subdivided into dorsomedial and ventromedial regions), the lateral PFC (subdivided into dorsolateral and ventrolateral regions), and the orbitofrontal cortex (OFC) (Kolk & Rakic, 2022). This brain region is highly interconnected with other cortical regions and other subcortical structures through serotonergic, noradrenergic, dopaminergic, and acetylcholinergic projections to these regions and/or structures. The density of fibers that utilize these neurotransmitters varies across each subdomain of the PFC. For example, the medial PFC has a higher concentration of cholinergic fibers compared to the other subdomains (Dembrow et al., 2014). Additionally, the types of receptors for each neurotransmitter may affect behavioral regulation, with varying levels of expression for each neurotransmitter in the PFC subdomains (Dembrow et al., 2014; Kolk & Rakic, 2022).

The PFC is crucial for behavioral regulation, specifically to adapt and cope effectively with challenging environments. Accordingly, this brain region underlies a set of cognitive processes that have been integrated and defined as executive functions. These functions comprise the ability to plan, initiate, make decisions, inhibit inappropriate responses, and cognitive flexibility, among others (Hall, 1993; Lezak, 1995). Therefore, from the very beginning, the PFC has been a focus of research when it comes to measuring or assessing abnormalities in human behavior, such as violence proneness or antisocial behavior.

Surgical reports from the end of the 19th to the early twentieth centuries concluded that after removing or damaging the PFC in humans and monkeys, those individuals did not show significant changes in behavior or intelligence. However, later evidence shifted the initial hypothesis that considered this brain region as “silent” to one that indicates damage in the PFC leads to significant alterations in goal-directed thought and behavior, now referred to as “frontal lobule syndrome” (Szczepanski & Knight, 2014), later refined to “dysexecutive syndrome” (Krch, 2011). That is, the concept evolved from a broad term encompassing a set of behavioral (e.g., disinhibition), affective (e.g., anhedonia, pseudopsychopathy, pseudodepression, among others), and cognitive symptoms to a more focused definition related to executive functioning, which includes a set of top-down cognitive processes (Diamond, 2013; Krch, 2011). Specifically, the behavior of individuals affected by PFC malfunctioning tends to be characterized by inflexibility, exhibiting serious difficulties when it comes to planning their behavior, with important alterations supervising and monitoring their behavior, problems anticipating consequences of their behavior and, consequently leading to risky decisions (Besnard et al., 2018; Lassaletta, & Bize, 2019). Therefore, it seems logical to conclude that some of these symptoms, partly due to PFC alterations, may characterize the behavior observed in violent individuals and criminals. Thus, the PFC could be viewed as a "brake" for emotional changes influenced by limbic structures, including the amygdala (Bertsch et al., 2020; Coccaro et al., 2011; Lischinsky & Lin, 2020).

Regarding the contribution of the PFC to cognitive processes that may influence violence intake or facilitation, it is important to highlight the different roles of each subdomain of the PFC. For example, the ventromedial PFC (VMPFC) tends to remain deactivated, especially during attention-demanding cognitive tasks. In fact, this deactivation correlates with better performance on attention-shifting tasks, which may help prioritize relevant stimuli and disengage from irrelevant ones (Fox et al., 2005; Mukahirwa et al., 2021). Conversely, the activation of the lateral PFC appears to be important in set-shifting tasks, while the activation of the OFC is crucial for adapting responses after receiving negative feedback (Friedman & Robbins, 2022). Thus, the contribution of the PFC cannot be solely understood in terms of behavioral disinhibition. It has been hypothesized that reactive and impulsive behaviors (or those mediated by emotions) could be explained by the roles of the VMPFC and dorsomedial PFC (DMPFC) in reward-based processes that influence decision-making, potentially leading to violence under certain circumstances (Blair, 2016; Dugré & Potvin, 2023).

The number of empirical research systematically studying the PFC functioning of violent individuals and criminals employing neuropsychological tests started rising in the second half of the twentieth century (Brower et al., 2001; Kandel & Freed, 1989; Morgan & Lilienfeld, 2000). Despite the vast heterogeneity of conclusions from these studies, much of the empirical research indicates that violent and antisocial individuals often score lower on certain tests measuring PFC functioning when compared to normative or non-violent individuals (Brower et al., 2001; Kandel & Freed, 1989; Morgan & Lilienfeld, 2000). All three reviews highlighted the importance of careful conclusions about the clear and strong link between PFC changes, particularly after injuries, and a tendency toward violence, regardless of the type of violence (e.g., reactive, proactive) or aggressor (e.g., persistent offenders, intimate partner violence, sexual aggressors), concretely, without considering moderating variables such as personality traits or mental disorders, among others. Additionally, it is crucial to be cautious, as neuropsychological tests may not always be reliable indicators of PFC functioning (Burgess & Stuss, 2017).

The incorporation and dissemination of neuroimaging techniques have supported the conclusions described above. Magnetic resonance imaging (MRI) allows for the non-invasive visualization of brain structure and function (Yen et al., 2023). Structural MRI has been used to measure the morphology, size, or location of brain regions, subcortical structures, and structural connectivity for clinical and research purposes (Symms et al., 2004). Meanwhile, functional MRI can effectively capture ongoing neural activity at different temporal scales, identifying regions and networks involved in various cognitive and emotional processes based on changes in brain blood flow. Other techniques, such as Positron Emission Tomography, Proton Magnetic Resonance Spectroscopy, and Single-Photon Emission Computed Tomography, assess brain metabolism, while magnetoencephalography measures the magnetic activity generated by neural activity (Yen et al., 2023). These functional techniques can be applied to measure brain activity in response to specific tasks as well as in resting conditions to assess intrinsic brain activity (resting stable functional connectivity).

Several seminal studies published in the mid-nineties of the twentieth century sparked interest in analyzing the PFC as a factor in violence, despite earlier attempts in 1949 to measure cortical functioning in murderers using electroencephalographic techniques (Stafford-Clark et al., 1949). One of the first published studies using magnetic resonance imaging (MRI) reported the existence of frontal dysfunctions in approximately 65% of a group of murderers sentenced to death (Blake et al., 1995). However, most conclusions were drawn from combining neuropsychological tests that measure frontal lobe functioning with electroencephalographical registers and magnetic resonance imaging (MRI). That is, not all men classified as exhibiting “frontal lobe symptoms” had focal PFC damages or alterations. Another contemporary study presented an ambitious longitudinal research project called the Vietnam Head Injury Study. Veterans with focal ventromedial PFC (VMPFC) lesions exhibited significantly higher levels of violence compared to individuals with lesions in other brain regions, including lesions in other PFC subregions, as well as compared to other individuals without brain damage (Grafman et al., 1996). Over the years, the accumulation of evidence from neuroimaging measurements led to the conclusion that PFC alterations might be specific to certain populations, such as violent individuals with high antisocial and psychopathic traits (Yang & Raine, 2009). These authors pointed out that antisocial behavior was associated with reductions in structure and function in the right OFC and left dorsolateral PFC (DLPFC). Thus, the available data did not provide a clear picture of which PFC alterations (morphological or functional) and regions might be generalizable to violence proneness.

Other reviews and meta-analyses in this field of research have revealed important inconsistencies across studies regarding the central role of PFC in explaining violence proneness. This suggests that some moderating variables (e.g., age of participants, type of brain analysis…) may contribute to explaining this association, as well as the varying contributions of different PFC subdomains to this kind of behaviors (Dugré et al., 2025; Nikolic et al., 2022; Raschle et al., 2015; Wang et al., 2024; Wong et al., 2019). All of this reinforces the need for a systematic review to clarify whether the presence of mental or personality disorders in violent individuals significantly explains PFC alterations compared to violent individuals without these conditions, while paying special attention to analytical approaches (case–control vs. dimensional) and neuroimaging modalities (e.g., fMRI, sMRI, PET). Furthermore, the heterogeneity across studies highlights the importance of analyzing whether comparisons of PFC subregions are associated with different forms of violence. In this regard, it has been recently stated that reactive (emotionally mediated) and proactive (cold-blooded) violence are differently related to PFC subregions (Romero-Martínez et al., 2022). Additionally, the application of non-invasive brain stimulation techniques to PFC subregions produces different effects on anger states and violent behavior (Romero-Martínez et al., 2025).

With all this in mind, the present systematic review was conducted to address this issue and aimed: (a) to summarize the main outcomes of neuroimaging research studies measuring the morphology and/or functioning of the PFC (total or subregions) in different samples of violent and/or criminal adults compared to non-violent individuals (or controls), and (b) to examine the potential association between the PFC (morphology and/or functioning) and violent behavior in adults. This second is complemented by a meta-analytic approach based on a few studies that provided data (correlation coefficients). Assessing both aims will help to clearly specify which samples of violent adults may exhibit considerable PFC alterations (e.g., with or without mental or personal disorders), guiding future research in this field to determine how to propose further investigations (e.g., focusing on PFC interconnections). It may also help identify alternative targets or modulatory variables (e.g., focusing on specific samples, other constructs related to violence, different brain regions, combinations with other types of treatment, among others) to address violence treatment or control. The conclusions may also help propose alternative intervention programs based on the development of specific forensic profiles for each subsample of violent individuals to properly and effectively reduce violence in certain types of adults.

## Methods

### Search strategy

The main guidelines established in the Preferred Reporting Items for Systematic Reviews and Meta-Analyses (PRISMA) (Hutton et al., 2015) were carefully followed to conduct this systematic review. In this sense, we applied a set of terms in the following multidisciplinary digital databases: Scielo, Scopus, Web of Knowledge, and PsycInfo. In fact, two of these databases (Scopus and Web of Knowledge) were selected according to the recommendations outlined by Bramer et al., (2017), signaling both as efficient databases for covering a wide range of relevant scientific literature. These databases provide some filters to reduce the number of entries, those were applied according to the inclusion and exclusion criteria established for our study. Accordingly, the following filters were applied to remove: “books”, “PhD”, “comment”, “note”, “editorial”, “erratum”, “book”, “book series”, “letter to the editor”, “conferences papers”, “conference proceeding”, “case reports”, “animal studies”, “systematic reviews”, “literature review”, “meta-analysis”, other languages different from “English and/or Spanish”, among others.

The terms applied for this systematic review and their combinations are described below: (prefrontal cortex OR dorsomedial cortex OR ventromedial cortex OR dorsolateral cortex OR ventrolateral cortex OR orbitofrontal cortex AND violence OR anger OR aggression OR hostility). Specifically, the algorithm employed to conduct this review was: ((prefrontal AND cortex AND violence) OR (prefrontal AND cortex AND anger) OR (prefrontal AND cortex AND aggression) OR (prefrontal AND cortex AND hostility) OR (dorsomedial AND cortex AND violence) OR (dorsomedial AND cortex AND anger) OR (dorsomedial AND cortex AND aggression) OR (dorsomedial AND cortex AND hostility) OR (ventromedial AND cortex AND violence) OR (ventromedial AND cortex AND anger) OR (ventromedial AND cortex AND aggression) OR (ventromedial AND cortex AND hostility) OR (dorsolateral AND cortex AND violence) OR (dorsolateral AND cortex AND anger) OR (dorsolateral AND cortex AND aggression) OR (dorsolateral AND cortex AND hostility) OR (ventrolateral AND cortex AND violence) OR (ventrolateral AND cortex AND anger) OR (ventrolateral AND cortex AND aggression) OR (ventrolateral AND cortex AND hostility) OR (orbitofrontal AND cortex AND violence) OR (orbitofrontal AND cortex AND anger) OR (orbitofrontal AND cortex AND aggression) OR (orbitofrontal AND cortex AND hostility)).

The search for published studies was conducted during November 2023 (from the 1 st to the 27th). Furthermore, potentially relevant articles were selected by applying a snowballing process based on the references of some articles included in our review.

The procedure to conducting this systematic review was also registered in OSF (Registration 10.17605/OSF.IO/ZAXT7).

### Inclusion criteria

**Fig. 1:**
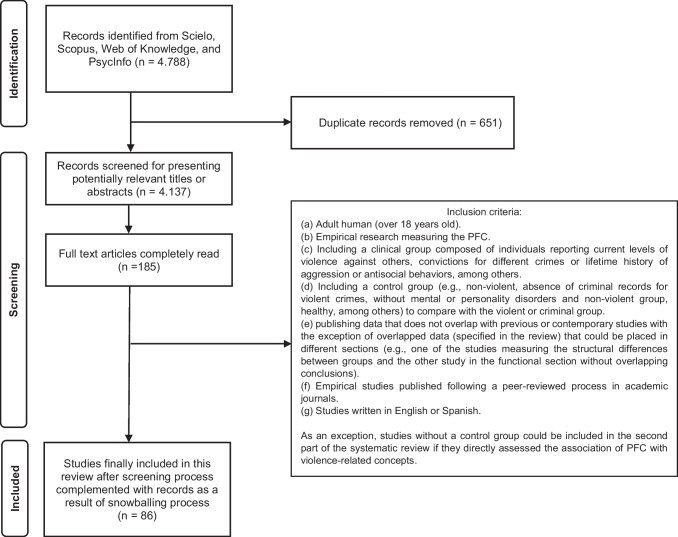
PRISMA flow chart of literature search with inclusion criteria

We established the inclusion criteria based on the recommendatins by McKenzie et al., (2019) for conducting systematic reviews and meta-analysis. In this sense, inclusion criteria for the systematic review were: (a) adult human (over 18 years old); (b) empirical research measuring the PFC; (c) including a clinical group composed of individuals reporting current levels of violence against others, convictions for different crimes or life history of aggression or antisocial behaviors, among others; (d) including a control group (e.g., non-violent, absence of criminal records for violent crimes, without mental or personality disorders and healthy, among others) to compare with the violent or criminal group; (e) publishing data that does not overlap with previous or contemporary studies with the exception of overlapped data (specified in the review) that could be placed in different sections (e.g., one of the studies measuring the structural differences between groups and the other study measuring the functional section without overlapping conclusions); (f) empirical studies published following a peer-reviewed process in academic journals; (g) studies written in English or Spanish. These criteria were essential for inclusion in the first and second parts of the systematic review. However, as an exception, studies without a control group could be included in the second part of the systematic review if they directly assessed the association of PFC (structure, activation, function or functional connectivity) with violence (e.g., previous history of violence, trait aggression, anger expression, antisocial and violent crimes, among others) (PRISMA flow diagram; Fig. 1).

### Exclusion criteria

Empirical studies published as case studies, letters to the editors, comments, errata, conference proceedings, preprints and gray literature were excluded. Additionally, empirical research which did not explicitly mention that adults included in their studies presented a life history of aggression, violent crimes or antisocial and violent behavior, specifically different forms of interpersonal violence, was also excluded. In this sense, studies measuring patients or individuals with self-injuries or suicidal ideation were removed. Studies which assessed group differences in self-reports measuring anger trait but did not indicate whether those participants had records regarding previous violent incidents were not considered. Furthermore, empirical studies that did not explicitly provide neuroimaging measurements of the PFC were excluded. Additionally, post-mortem studies or those involving patients with brain damage were also discarded from this review. This is because it is extremely difficult to determine which patients have specific brain damage to the PFC without noting whether other brain regions were also affected. This also applied to post-mortem studies which did not provide enough information regarding cause of death and its effects on brain integrity. Lastly, we paid special attention to overlapping data across studies, discarding research that presented the same data in the same section (e.g., gray matter volume measurements in violent men without a clinical diagnosis who were later diagnosed with psychopathy). However, studies that presented overlapping data but belonged to different sections (e.g., structural measurements and functional connectivity) were included, with explicit notes on the data overlap. This approach facilitated transparency in interpreting the results of this review.

### Quality assessment

Based on the established in the National Institutes of Health (NIH), (2021) and previously published systematic reviews (Romero-Martínez et al., 2022; 2024a) to assess the potential risk of bias when interpreting the data of the included empirical research, we established a set of quality criteria to assess the studies included in our systematic review. In this sense, nine questions were raised to be answered with a dichotomous answer (Yes/No) indicating whether each criterion was met or not in each study. The interpretation of the risk of bias was as follows: three or fewer statements answered with “no” were classified as “low risk of bias”. Empirical studies that neglected four to six statements, answered with “no” were classified as “moderate risk of bias”, and those neglecting seven or more criteria were classified as “high risk of bias” (See Supplementary Table 1). The degree of agreement between both independent evaluators needs to be higher than.80 to evaluate the risk of bias. Each of the assessed domains are described below:

- ***Objectives and hypotheses***
  1. Authors employ the introduction to enumerate and clearly define the objectives and hypothesis based on previous scientific literature by citing references and specifying the main outcomes (e.g., PFC subregion and their differences between groups, expected association between variables, etc.). Being considered “no” when authors apply general statements without specifying.
- ***Sample***
  - A theoretical justification of the number of participants in the study (e.g., exceptionality of the mental disorder, reduced number of comparisons, among others) is calculated or provided.
  - Sample size is equal to or higher than 50 participants per group.
  - Inclusion criteria are specified and clearly stated for each group or sample included in the study.
- ***Procedure***
  - Procedure is clearly defined (e.g., time frame, experimental phases, instruments employed, etc.).
- ***Results***
  - Absence of differences between groups in potential confounding variables (e.g., number of participants per group, sociodemographic differences such as age, educational level, among others) is clearly reported.
  - Potential confounding variables are covariated.
  - Mean values and standard deviations of relevant variables of the study are provided (e.g., published or as supplementary material).
  - Authors controlled the effect of multiple comparisons.

### Data extraction and analysis

Two of three authors conducted the article selection, with an interrater agreement above 85%. Discrepancies between raters (e.g., meeting the inclusion criteria or not) were discussed between them and by asking the third author of the study.

### Data analysis

The new version of RefWorks was used for reference management (ProQuest, 2016).

The Meta-Essentials for correlational data 1.5 (Suurmond et al., 2017) was used for a complementary analysis of the second aim of the systematic review. This was done when there was enough data to calculate the association between PFC and different forms of violence. To achieve this, a minimum of two different articles was required to provide the necessary data, as recommended by Ryan, (2016). Tau-squared (Tau^2^), Cochran's Q (Q), and I-squared (I^2^) were used to assess the presence of heterogeneity across the included studies. Regarding the interpretation of these values, Tau^2^ is the estimated variance of the effect sizes in various research studies. Accordingly, it measures the degree of genuine dispersion of effects. A value of 0 represents an absence of variability across studies, while a value higher than 0 indicates a dispersion of effect sizes among the included studies. Furthermore, a high Q value together with a pQ value lower than 0.05 might indicate the presence of heterogeneity across studies. Additionally, I^2^ is the percentage of total variability across studies; the higher the I^2^, the higher the variability across studies (e.g., higher than 40). Egger's test was used to quantify the publication bias in the meta-analysis, with a significant intercept suggesting the presence of publication bias (Egger et al., 1997). Moreover, we also included the Begg & Mazumdar Z value, which is a standardized test statistic, with a significant Z value indicating the presence of publication bias (Begg et al., 1994).

## Results

### Selection of studies

Applying the algorithm described above by combining the terms selected for this study and applying, when possible, the above-described filters yielded a total of 4788 sources across the four databases. The analysis of duplicates in RefWorks was then applied resulting in the removal of 651 references. The remaining 4137 articles were screened based on title or abstract. After screening the articles for potentially relevant terms (e.g., PFC, aggressive behavior, violence, among others), a total of 2914 articles were selected. From the relevant scientific literature, 185 articles were selected, which were completely read resulting in a total of 82 to finally be included in the systematic review. Additionally, the snowballing process allowed us to further select a total of 4 articles. Therefore, this systematic review was conducted based on a total of 86 empirical articles (see the flowchart described in Fig. 1).

### Systematic review

Individuals without mental or personality disorders with a history of violent behaviors and/or criminal offending (e.g., committed violent crimes, perpetrated violent attacks against others).

### Murderers

This section included three studies conducted with men convicted of homicide compared to control participants not accused of any crimes. One of them addressed the structural section, the other two the functional section under resting conditions. It could be concluded that murderers (men and women) exhibited a bilateral reduction of GMV in the OFC compared to controls (Lam et al., 2017). Nevertheless, studies also concluded that murderers of both genders also showed higher cortical thickness in the medial OFC (bilaterally) compared to controls. Therefore, murderers exhibited reductions in volume in the OFC, but higher cortical thickness in this PFC subregion.

Regarding PFC functioning in individuals convicted of murder, both genders exhibited a lower glucose metabolism in the medial PFC (bilaterally) and lateral left PFC under resting conditions compared to the control group (Raine et al., 1994, 1997). However, not all conclusions between both studies were homogeneous given that certain discrepancies existed between both. Concretely, Raine et al., (1994) demonstrated the existence of a reduced medial (bilateral) and left lateral PFC glucose metabolism in murderers when compared to controls. However, their next study went beyond the reduced glucose metabolism in the medial PFC (bilaterally), and also observed a bilateral reduction in glucose metabolism in the lateral PFC (Raine et al., 1997) (Table 1). Given that both studies were conducted by the same research team, the discrepancy between them may be attributed to the smaller sample size used and the considerable heterogeneity across samples due to the limited number of variables included in the study for defining the samples.

**Table 1:** Summary of characteristics of the studies assessing group differences in prefrontal cortex in murderers and violent offenders without mental or personality disorders, alphabetically ordered by first author’s surname

Authors	Sample	Age	Gender	Educational level and handedness	Neuroimaging characteristics	Laboratory task	ROI and covariates	Results
**Murderer**								
**Structural**								
Lam et al., 2017	Murderers (*n* = 23) Controls – not accused of any crimes (*n* = 44)	36.91 ± 14.36 32.61 ± 10.18	83% ♂ 84% ♂	-	MRI (gray matter volume and cortical thickness), 1.5 T	-	ROI: OFC and striatum Covariates: age, IQ, sex, socioeconomic status, schizophrenia diagnosis and whole brain volume	Murderer < GMV in lateral OFC (left and right) than controls Murderer > cortical thickness medial OFC than controls Absence differences in lateral OFC GMV and lateral OFC cortical thickness
**Functional**								
Raine et al., 1994	Murderer (*n* = 22) Controls – not accused of any crimes (*n* = 22)	35.4 ± 10.4 34.2 ± 10.9	91% ♂ 91% ♂	9% right-handed -	PET-FDG	Resting	ROI: PFC (superior frontal gyrus, middle frontal gyrus, and inferior frontal gyrus), posterior (non-prefrontal) frontal cortex, bilateral temporal (superior, middle, inferior, posterior) and parietal (postcentral, supramarginal, superior parietal lobule, and angular gyrus) Covariates: - Groups matched for age and gender	Reduced medial PFC (bilateral) glucose metabolism in the murderer. Furthermore, reduced left lateral PFC glucose metabolism in the murderer
Raine et al., 1997	Murderers (*n* = 41) Controls – not accused of any crimes (*n* = 41)	34.3 ± 10.1 31.7 ± 10.3	95% ♂ 77% ♂	86% right-handed	PET-FDG	Resting	ROI: PFC (superior frontal gyrus, middle frontal gyrus, and inferior frontal gyrus), bilateral temporal (superior, middle, inferior, and posterior), parietal (postcentral, supramarginal, superior parietal lobule, and angular gyrus), and occipital (area 19, area 17 superior, area 17 inferior, and area 18) Covariates: - Groups matched for age and gender	Lower glucose metabolism lateral and medial PFC (bilaterally) in murderers in comparison with controls
**Offenders and individuals reporting high lifetime history of aggression against others**								
**Structural**								
Coccaro et al., 2018	Individuals with LHA (e.g., high number of times engaged in overt aggressive behavior over their life) (*n* = 35) Individuals without LHA (*n* = 36)	-	-	-	MRI (gray matter volumen), 3 T	-	ROI: whole brain analysis Covariates: age, gender, race, socioeconomic status, impulsivity, history of syndrome disorder, and history of personality disorder	GMV in individuals with LHA was lower in mPFC and lPFC than individuals without LHA
Leutgeb et al., 2015	Violent offenders (*n* = 40) Non-offender controls (*n* = 37)	38.1 ± 12.0 36.7 ± 9.6	100% ♂	11.4 ± 1.9 11.6 ± 1.4	MRI (gray matter volume), 3 T	-	ROI: DLPFC, DMPFC, OFC amygdala, insula, caudate nucleus, pallidum, putamen, cerebellar hemispheres, vermis, and SMA Groups matched for whole brain volume	Offenders show lower GMV in the DMPFC (bilaterally) than controls
Leutgeb et al., 2016	Violent offenders (*n* = 31) Non-offender controls (*n* = 30)	36.8 ± 12.0 35.1 ± 9.0	100% ♂	11.3 ± 1.7 11.6 ± 1.0	MRI (gray matter volume) + rsFC, 3 T	-	ROI: whole brain analysis Covariates: - Groups matched for educational level, handedness and free of drug use	Offenders presented lower GMV in DLPFC (bilaterally) than controls
Marín-Morales et al., 2022	IPV offenders (*n* = 26) Other criminals (*n* = 29) Non-offender controls (*n* = 30)	41.19 ± 9.71 39.77 ± 10.79 38.28 ± 8.24	100% ♂	9.19 ± 4.3 9.5 ± 3.61 9.86 ± 2.44	MRI (gray matter volume), 3 T	-	ROI: dlPFC, vmPFC, dorsal and ventral ACC, Nucleus Accumbens, insula, and amygdala Covariates: age, severity of drug consumption, and total intracranial volume	Absence of group differences between groups in DLPFC or VMPFC
Nummenmaa et al., 2021	Violent offenders (*n* = 19) Healthy controls (*n* = 19) Community (*n* = 100)	31.16 ± 6.49 28.53 ± 7.69 31.14 ± 9.31	100% ♂ 100% ♂ 49% ♂	-	MRI (gray matter density) + PET, 3 T	Viewing violent scenes	ROI: OFC Covariates: primary and secondary psychopathy	Offenders lower GMD in OFC than healthy controls Offenders presented higher activation in OFC than healthy controls viewing scenes
Schiffer et al., 2011	Violent offenders (*n* = 12) Violent offenders + drugs (*n* = 12) Non-offender controls (*n* = 14) Non-offender controls + drugs (*n* = 13)	37.4 ± 10.6 36.4 ± 5.5 36.7 ± 11.4 37.3 ± 7.9	100% ♂	9.55 ± 1.21 9.50 ± 0.97 9.93 ± 0.99 9.69 ± 1.44 100% right-handed	MRI (gray matter volume), 1.5-T	-	ROI: whole brain analysis Covariates: age, IQ, total gray matter volumes, and life history of alcohol and drug misuse	No differences were detected in GMV of the PFC or OFC between the violent offenders and the nonoffenders
Tiihonen et al., 2008	Violent offenders (*n* = 26) Non-offender controls (*n* = 25)	32.5 ± 8.4 34.6 ± 10.8	100% ♂	-	MRI (gray matter volume), 1 T	-	ROI: whole brain analysis Covariates: total intracranial volume and age	Offenders presented lower GMV in OFC than controls
Varkevisser et al., 2021	Violent veterans (*n* = 29) Non-violent veterans (*n* = 30)	36.28 ± 1.17 34.53 ± 1.39	100% ♂	-	MRI (cortical thickness), 3 T	-	ROI: dorsolateral prefrontal cortex, orbitofrontal cortex, and anterior cingulate cortex Covariates: total intracranial volume and age	Absence differences in cortical thickness of the left or right OFC, ACC, and/or DLPFC
**Functional**								
**Resting condition**								
Amaoui et al., 2022	IPV offenders (*n* = 26) Other criminals (*n* = 29) Non-offender controls (*n* = 29)	41.19 ± 9.71 38.97 ± 11.05 38.28 ± 8.54	100% ♂	-	rsFC, 3-T	rsFC	ROI: amygdala, PFC, striatum and insula Covariates: drug severity and emotional regulation	IPV > Controls Left VLPFC with Brainstem, Middle Temporal area and hippocampus; Left DLPFC cortex with putamen-caudate IPV < Controls Right VLPFC with sensorimotor area, premotor area, intraparietal sulcus and occipital area rsFC was not tested between other criminals and controls
Leutgeb et al., 2016	Violent offenders (*n* = 31) Non-offender controls (*n* = 30)	36.8 ± 12.0 35.1 ± 9.0	100% ♂	11.3 ± 1.7 11.6 ± 1.0	fMRI, 3 T	rsFC	ROI: OFC, DLPFC, amygdala, cerebellum, vermis Covariates: - Groups matched for educational level, handedness and free of drug use	Offenders presented higher rsFC between the left and the right DLPFC than controls Offenders presented lower rsFC between the left/right cerebellar hemisphere with the left/right OFC Offenders presented lower rsFC between vermis with left OFC
Varkevisser et al., 2017	Violent veterans (*n* = 28) Non-violent veterans (*n* = 30)	36.54 ± 6.27 34.53 ± 7.59	100% ♂	68% middle 53% middle	fMRI, 3 T	rsFC	ROI: BLA, CeM, and ACC Covariates: - Groups matched for age, educational level, number of deployments and military rank	Reduced functional connectivity between the (bilateral) BLA and left DLPFC in the impulsive aggression group, relative to combat controls
Wolfs et al., 2023	Violent veterans (*n* = 19) Non-violent veterans (*n* = 22)	35.0 29.5	100% ♂	-	fMRI, 3 T	rsFC	ROI: whole-brain analysis Covariates: - Groups matched for age, number of deployments and time of deployment	Veterans showed diminished rsFC between deep cerebellar nuclei and left OFC in comparison with controls (significant after controlling medication use)
Booij et al., 2010	Individuals with LHA (e.g., physical aggression) (*n* = 8) Individuals without LHA (*n* = 18)	27.1 ± 0.7	100% ♂	-	PET, 1.5 T	Resting	ROI: OFC, caudate and hippocampus Covariates: self-reported impulsivity	Violent individuals presented lower 5-HT synthesis bilaterally in the OFC in comparison with low violent individuals
Critchley et al., 2000	Violent forensic individuals (*n* = 10) Non-violent forensic individuals (*n* = 8)	23 ± 5.0 29 ± 5.0	90% ♂ 75% ♂	-	Proton Magnetic Resonance Spectroscopy, 1.5 T	Resting	ROI: Medial PFC and medial temporal lobe Covariates: age and IQ	Violent patients had lower concentrations of NAA and Cr1PCr in medial PFC than controls Violent patient group, frequency of observed violence to others correlated significantly with prefrontal lobe NAA concentration (*r* = -.72, *p* <.05)
da Cunha-Bang et al., 2017a	Violent offenders (*n* = 19) Non-offender controls (*n* = 24)	31.4 ± 8.7 32.4 ± 10.7	100% ♂	8.9 ± 2.5 11.3 ± 1.1 education	PET	Resting	ROI: anterior cingulate cortex, OFC, and striatum Covariates: age, IQ and injected mass per kilogram of body weight	Group comparisons revealed no difference in 5-HT1BR binding or gray matter volumes of the striatum, ACC, or OFC
George et al., 2004	IPV + alcohol (*n* = 8) Non-offenders + alcohol (*n* = 11) Non-offender controls (*n* = 10)	32.9 ± 6.2 39.8 ± 7.2 38.1 ± 9.5	100% ♂	-	PET	Resting	ROI: thalamus, posterior OFC, amygdala, basal forebrain, hypothalamus, anterior cingulate cortex, posterior cingulate cortex and caudate Covariates: - Groups matched for age	Absence differences in regional cerebral metabolic rate of glucose uptake in right posterior OFC and left posterior OFC
Meyer et al., 2008	Individuals with LHA (e.g., assault with blunt objects, physical fights, etc.) (*n* = 16) Individuals without LHA (*n* = 16)	29.31 ± 6.26 29.06 ± 6.17	56% ♂ 63% ♂	-	PET, 1.5 T	Resting	ROI: middle frontal gyrus, DLPFC, OFC, posterior medial temporal gyrus and anterior cingulate Covariates: age	PFC 5-HT2A binding potential in DLPFC was significantly higher in participants with more severe impulsivity and aggression (modulated for age)
**Laboratory tasks**								
Alia-Klein et al., 2014	Individuals with LHA (e.g., physical fights in the last year) (*n* = 12) Individuals without LHA (*n* = 13)	24.09 ± 0.80 25.40 ± 0.80	100% ♂	0.86 ± 0.07 0.92 ± 0.02	PET-FDG	3 conditions: resting baseline, violent scenes, non-violent emotional scenes	ROI: whole brain analysis Covariates: - Groups matched for age, handedness, socioeconomic status, IQ and depressive symptoms	Resting baseline → Aggressive < controls (OFC) Violent scenes → Aggressive < controls (OFC) Non-violent emotional scenes → absence group differences
Chester and DeWall, 2019	IPV perpetrators (*n* = 23) Non-IPV perpetrators (*n* = 38)	From 18 to 22	17% ♂ 38% ♂	100% university students 100% right-handed	fMRI, 3 T	Taylor Aggression Paradigm	ROI: Medial PFC (dorsal and ventral) Covariates: gender	IPV perpetrators showed less ventral-than-dorsal MPFC reactivity to provocation than non-IPV perpetrators More ventral-than dorsal MPFC reactivity was associated with a reduced likelihood of IPV perpetration, OR < 0.01, B = − 10.89, SE = 5.24, Χ(1, 58) = 4.33, *p* =.038
da Cunha-Bang et al., 2017b (overlapped with da Cunha-Bang et al., 2017a)	Incarcerated violent offenders (*n* = 18) Non-offender controls (*n* = 26)	31.8 ± 8.8 29.6 ± 9.2	100% ♂	8.9 ± 2.6 11.6 ± 0.8	fMRI, 3 T	12-min session of the PSAP and the monetary reward	ROI: amygdala, striatum (i.e. caudate and putamen) and the PFC (including anterior cingulate cortex) Covariates: age and group	The groups did not differ significantly in response to provocations within the PFC
Heesink et al., 2018	Violent veterans (*n* = 28) Non-violent veterans (*n* = 28)	36.29 ± 6.43 34.21 ± 7.75	100% ♂	4.21 ± 0.63 4.21 ± 0.79	fMRI, 3 T	96 pictures from the IAPS. These pictures elicit general emotional experience	ROI: amygdala and the dACC with other areas of the brain in relation to the task Covariates: - Groups matched for age, educational level and number of deployments	Violent veterans showed weaker connectivity between dACC with medial PFC during the presentation of positive stimuli Failed to find association between dACC with MidOFC
Hofhansel et al., 2023	Violent offenders (*n* = 19) Non-offender controls (*n* = 12)	34.16 ± 9.51 33.50 ± 9.03	100% ♂	10.50 ± 0.86 13.27 ± 2.69	fMRI, 3 T	Anger frustration paradigm	ROI: 36 brain structures Covariates: - Groups matched for age	Offenders presented less connectivity between the dorsomedial prefrontal cortex (DMPFC) and the left MTG than controls
Lee et al., 2009	IPV (*n* = 10) Non-IPV (*n* = 13)	43.80 ± 5.10 47.08 ± 6.25	100% ♂	10.70 ± 4.11 9.85 ± 2.04	fMRI, 3 T	Picture viewing task (affective pictures)	ROI: whole brain analysis Covariates: - Groups matched for age, educational level, years of marriage, employment status and nonverbal IQ	Positive pictures: IPV > activation controls (right inferior orbitofrontal gyrus) Controls > activation IPV (right superior orbitofrontal gyrus) Aggressive female victims (IPV > Controls) activation right precentral gyrus
Lee et al., 2008	IPV (*n* = 10) Non-IPV (*n* = 13)	-	100% ♂	-	fMRI, 3 T	Cognitive and Emotional Stroop	ROI: whole brain analysis Covariates: -	IPV showed less activation of the left middle frontal gyrus when aggressive words (Emotional Stroop) Absence of differences in cognitive Stroop
Nummenmaa et al., 2021	Violent offenders (*n* = 19) Healthy controls (*n* = 19) Community (*n* = 100)	31.16 ± 6.49 28.53 ± 7.69 31.14 ± 9.31	100% ♂ 100% ♂ 49% ♂	-	MRI (gray matter density) + PET, 3 T	Viewing violent scenes	ROI: OFC Covariates: - Groups matched for age and body mass index	Offenders presented higher activation in OFC than controls viewing violent scenes
Pardini & Phillips, 2010	Individuals with LHA (e.g., often involved in moderate/serious violent acts) (*n* = 22) Individuals without LHA (*n* = 20)	26.18 ± 1.04 26.84 ± 0.91	100% ♂	-	fMRI, 3 T	Facial task	ROI: the amygdala, OFC, dorsomedial prefrontal cortex and medial prefrontal cortex Covariates: - Groups matched for IQ	Violent men presented lower activation in left DMPFC than controls (significant after controlling age, past year hard drug use, positive urine screen for marijuana and ADHD symptoms)
Prehn et al., 2013	Offender emotionally hyporeactive (*n* = 11) Offender emotionally hyperreactive (*n* = 12) Non-offender controls (*n* = 13)	27.55 ± 5.57 27.75 ± 10.24 26.62 ± 8.40	100% ♂	-	fMRI, 1.5 T	Risk-avoidant and risk-seeking decision making	ROI: whole brain analysis Covariates: - Groups matched for age and IQ	Neural activity preceding all choices → Absence group differences in PFC between groups
Siep et al., 2019	Violent offenders (*n* = 18) Non-offender controls (*n* = 18)	35.17 ± 7.12 37.06 ± 15.24		83% basic educ 56% basic educ	fMRI, 3 T	Emotional provocation or engagement	ROI: bilateral amygdala with the rest of the brain regions Covariates: - Groups matched for age and educational level	Offenders presented a decrease in left medial PFC connectivity with the left amygdala from pre- to post-task, whereas controls showed an increase in the connectivity between both brain regions. Post-hoc simple-effects analysis revealed that the largest difference between groups were pre-task, with a larger left medial prefrontal connectivity with the left amygdala in offenders in comparison with controls. Post-task the difference in left medial prefrontal connectivity with the left amygdala seed between groups was no longer significant (*p *=.34) Results showed a significant relationship between activity in the medial PFC connectivity during the pre-task scan and (reactive as well as proactive) aggression (*r* =.36, *p* <.05 for reactive aggression and *r* =.45, *p* <.01 for proactive aggression)
Spoont et al., 2010	Individuals with LHA (e.g., to have at least one physically violent act within the prior year, history of repetitive violent behaviour, etc.) (*n* = 8) Individuals without LHA (*n* = 8)	44.25 ± 9.3 42.4 ± 9.2	100% ♂	13.3 ± 1.5 15 ± 1.8 75% right-handed	PET	Autobiographical scripts (anger vs neutral)	ROI: whole brain analysis Covariates: - Groups matched for age, educational level and history of chemical dependency	Absence of differences during resting condition or other conditions in PFC between groups
Tonnaer et al., 2017	Violent offenders (*n* = 16) Non-offender controls (*n* = 18)	35.81 ± 7.17 34.39 ± 13.37	100% ♂	89% secondary 88% secondary	fMRI, 3 T	Anger provocation and regulation task	ROI: whole brain analysis Covariates: - Groups matched for age, educational level and handedness	The left VLPFC showed stronger activity for the violent offenders during anger provocation compared to the controls Violent offenders presented higher left VLPFC activation during anger induction than during distraction. Controls showed the opposite pattern Violent offenders presented higher left DLPFC activation during anger or happy induction than during distraction in comparison with controls

*Note*: *ADHD* Attention-Deficit/Hyperactivity Disorder (ADHD), *dACC* dorsal anterior cingulate cortex, *DLPFC* dorsolateral prefrontal cortex, *DMPFC* dorsomedial prefrontal cortex, *FDG* F-fluorodeoxyglucose, *fMRI* functional magnetic resonance imaging, *GMD* gray matter density, *GMV* gray matter volume, *IAPS* international affective picture system, *IPV* intimate partner violence, *IQ* intelligence quotient, *LHA* life history of aggression, *MidOFC* middle orbitofrontal cortex, *MRI* magnetic resonance imaging, *MTG* middle temporal gyrus, *OFC* orbitofrontal cortex, *PET* positron emission tomography, *PFC* prefrontal cortex, *rCBF* regional cerebral blood flow, *SMA* supplementary motor area, *VLPFC* ventrolateral prefrontal cortex, *VMPFC* ventromedial prefrontal cortex

### Offenders who committed violent crimes against others (violent offenders)) other than murder

From the eight articles included in the violent offenders’ section measuring structural PFC differences between these individuals and controls not accused of any crimes (or non-offenders), five of them reported significant differences (Coccaro et al., 2018; Leutgeb et al., 2015, 2016; Nummenmaa et al., 2021; Tiihonen et al., 2008). That is, 63% of the studies highlighted that groups differed in PFC morphology. In all cases, offenders who committed violent crimes other than murder showed lower GMV and gray matter density (GMD) in specific PFC subregions. However, those studies differed in signaling which regions showed this reduction when compared to controls, concluding that violent offenders exhibited lower GMV in the medial PFC (Coccaro et al., 2018) or more specifically in the DMPFC (Leutgeb et al., 2015) and lateral PFC (Coccaro et al., 2018) or even more precisely in the DLPFC bilaterally in comparison with the control group (Leutgeb et al., 2016). The other two studies concluded that the OFC of violent offenders presented reduced volume and density compared to controls (Nummenmaa et al., 2021; Tiihonen et al., 2008).

The analyses of resting state functional connectivity (rsFC) comprised four studies (Amaoui et al., 2022; Leutgeb et al., 2016; Varkevisser et al., 2017; Wolfs et al., 2023), which offered a varied picture of the functional connectivity. For example, two of them focused on the functional connectivity between the OFC and the cerebellum. In this sense, violent offenders (men convicted of intimate partner violence, other types of violent offenses against others, etc.) showed lower rsFC between the vermis and deep cerebellar nuclei and the left OFC (Leutgeb et al., 2016; Wolfs et al., 2023). Additionally, violent offenders also showed lower rsFC between the cerebellar hemisphere and the OFC (bilaterally) (Leutgeb et al., 2016). Other PFC regions also exhibited differential activation with other brain regions in violent individuals compared to controls. Specifically, violent offenders exhibited reduced rsFC between the left DLPFC and the basolateral amygdala (bilaterally) compared to controls or non-offenders (Varkevisser et al., 2017). Conversely, violent offenders also showed higher rsFC between the left and the right DLPFC compared to controls (Leutgeb et al., 2016) and a heightened rsFC between the left DLPFC and the putamen-caudate (Amaiou et al., 2022). Lastly, violent offenders also exhibited a heightened rsFC between the left VLPFC and brainstem, middle temporal area and hippocampus as well as reduced rsFC between the right VLPFC and the sensorimotor area, premotor area, intraparietal sulcus and occipital area (Amaiou et al., 2022).

The use of PET techniques during resting periods in violent offenders as well as individuals with a history of persistent attacks against others, such as assault with blunt objects or physical fights was measured in five studies (Booij et al., 2010; Critchley et al., 2000; da Cunha-Bang et al., 2017a; George et al., 2004; Meyer et al., 2008), with three of them failing to report group differences (Chang et al., 2017; da Cunha-Bang et al., 2017a; George et al., 2004). Offenders did not differ from non-offender controls in 5-HT1BR binding in the OFC (da Cunha-Bang et al., 2017a), they showed lower 5-HT synthesis bilaterally in the OFC (Booij et al., 2010) and PFC 5-HT2A binding potential in the DLPFC, especially in younger violent individuals (Meyer et al., 2008) when compared to the control groups. Conversely, older offenders of both genders showed higher 5-HT2A levels than controls (Meyer et al., 2008). Nonetheless, no differences in regional cerebral metabolic rate of glucose in the OFC were found (George et al., 2004). Lastly, violent individuals also exhibited lower concentrations of N-acetylaspartate (NAA) and creatine phosphocreatine (Cr1PCr) in the medial PFC compared to controls (Critchley et al., 2000).

The analysis of group differences when participants were submitted to different laboratory tasks allowed us to include a total of 12 articles (Alia-Klein et al., 2014; Chester and DeWall, 2019; da Cunha-Bang et al., 2017b; Heesink et al., 2018; Hofhansel et al., 2023; Lee et al., 2008; Nummenmaa et al., 2021; Pardini & Phillips, 2010; Prehn et al., 2013; Siep et al., 2019; Spoont et al., 2010; Tonnaer et al., 2017). From all the studies, only three (25%) failed to report group differences in PFC functioning in response to different laboratory tasks (da Cunha-Bang et al., 2017b; Prehn et al., 2013; Spoont et al., 2010), which were mostly conducted with men, except for two studies that also included women (Chester & DeWall, 2019; Nummenmaa et al., 2021). The remaining studies showed varied findings. In fact, two groups of conclusions could be established based on the tasks used to measure responses to emotional stimuli (e.g., pictures of smiling babies or skulls) or those aimed at evoking anger. Regarding exposure to emotional images, violent offenders or individuals reporting a lifetime history of aggression against others exhibited lower connectivity between the medial PFC and dorsal anterior cingulate cortex (dACC) during the presentation of positive images compared to controls (Heesink et al., 2018). Additionally, other groups of violent offenders convicted of intimate partner violence (IPV) showed higher activation of the right inferior OF gyrus and lower activation of the right superior OF gyrus when viewing positive pictures compared to controls (Lee et al., 2008). Another study found that when violent offenders cope with emotional pictures (e.g., facial emotional expressions), they show lower activation in the medial PFC, specifically in the left DMPFC, compared to controls (Pardini & Phillips, 2010). However, there were inconsistencies regarding OFC activity in response to violent content. In this regard, Alia-Klein et al., (2014) concluded that men often involved in physical fights in the last year exhibited lower OFC activation during the presentation of violent scenes compared to controls, while Nummenmaa et al., (2021) reported the opposite. Supporting the findings of Alia-Klein et al., (2014), they concluded that violent offenders showed lower OFC activation compared to controls when preparing to respond to the laboratory task.

The rest of the studies focused on the induction of anger in both groups. In this case, violent offenders, especially men convicted of IPV perpetration, showed less VMPFC reactivity to provocation than controls (Chester & DeWall, 2019). Another study provided a detailed description of PFC activation during each phase of an anger frustration task. In this sense, violent offenders exhibited higher left VLPFC and DLPFC activity during anger engagement compared to controls. They also exhibited lower activation in the left VLPFC during a distraction period compared to controls. In fact, controls exhibited the opposite pattern of activation of VLPFC during anger engagement and distraction period (Tonnaer et al., 2017). This was complemented by subsequent research that measured functional connectivity of the PFC and other brain regions in response to laboratory anger induction tasks. Accordingly, violent offenders exhibited less connectivity between the DMPFC and the left middle temporal gyrus, as well as between the left medial PFC and the left amygdala during a frustrating anger task compared to controls (Hofhansel et al., 2023; Siep et al., 2019). Conversely, controls showed an increase in connectivity between the left medial PFC and the left amygdala (Siep et al., 2019) (Table 1).

### Individuals with mental or personality disorders (e.g., characterized by high anger expression-out, anger-prone) and a history of violent behaviors and/or criminal offending (e.g., who have committed violent crimes, who perpetrated violent attacks against others)

#### Patients with schizophrenia who committed violent crimes or violently assaulted others

This section included a total of nine articles combining men and women, although samples were mostly composed of men. From the included research, three focused on studying structural differences between individuals with schizophrenia reporting a high prevalence of violent attacks against others using control groups of healthy individuals without violent histories (Barkataki et al., 2006; Storvestre et al., 2019, Yang et al., 2010). In this sense, two of the studies failed to report structural PFC differences between groups (Barkataki et al., 2006; Yang et al., 2010). The only study that reported significant differences between groups found that violent individuals with schizophrenia exhibited an increased gyrification in the right lateral OFC compared to controls (Storvestre et al., 2019). That is, only 34% of the studies pointed out the existence of significant differences between groups.

The analysis during resting conditions was only conducted by one study. This study measured the metabolic glucose rate in PFC regions, failing to report group differences between two groups of individuals with schizophrenia (with and without violence) and a control group (Wong et al., 1997).

The other five studies provided heterogeneous conclusions, based on the type of task employed (Fortier et al., 2023; Joyal et al., 2007; Kumari et al., 2009; Schiffer et al., 2017; Tikàsz et al., 2018). On the one hand, it is possible to group those applying emotional processing (Schiffer et al., 2017; Tikàsz et al., 2018) and on the other, those employing cognitive tasks such as go/no go measuring inhibitory control and divided attention (Joyal et al., 2007; Kumari et al., 2006). Except for the case of Kumari et al., (2006), all of them were able to report differences between groups. Studies measuring PFC functioning during emotional tasks concluded that individuals with schizophrenia and antisocial personality disorder who had a history of violent behaviors exhibited higher activity in the medial PFC and left VLPFC compared to controls. However, in the case of violent patients with schizophrenia without antisocial personality disorder there were no differences in their PFC activation compared to controls (Schiffer et al., 2017). While the performance in the inhibiting task with emotional stimuli (angry faces) showed a decrease in activation in the right DLPFC in patients with schizophrenia who perpetrated violent attacks against others compared to controls (Tikàsz et al., 2018), the application of the inhibiting task without emotional stimuli revealed that violent patients with schizophrenia had reduced OFC activation (Joyal et al., 2007). Nevertheless, another study failed to report significant group differences between violent patients with schizophrenia and controls at the DLPFC level (Kumari et al., 2006) (Table 2).

**Table 2:** Summary of characteristics of the studies assessing group differences in prefrontal cortex in individuals with mental disorders and history of violent behaviors, alphabetically ordered by first author’s surname

Authors	Sample	Age	Gender	Educational level	Neuroimaging characteristics	Laboratory task	ROI	Results
**SZ**								
**Structural**								
Barkataki et al., 2006	SZ with LHA (e.g., fatal or near fatal act of violence against another) (*n* = 13) SZ without LHA (*n* = 13) Healthy controls (*n* = 15)	34.46 ± 4.94 34.47 ± 7.49 32.13 ± 7.47	100% ♂	-	MRI (gray matter volume), 1.5 T	-	ROI: whole brain analysis Covariates: whole brain volume	Absence of group differences in PFC
Storvestre et al., 2019	SZ with LHA (e.g., history of severe violence such as murder, attempted murder, or violent assault) (*n* = 11) SZ without LHA (*n* = 17) Healthy controls (*n* = 19)	33.2 ± 9.0 34.3 ± 7.4 33.2 ± 9.1	91% 94% 89%	10.4 ± 1.9 12.4 ± 2.6 13.7 ± 1.6	MRI (gyrification and cortical thickness), 3 T	-	ROI: whole brain analysis Covariates: age and sex	SZ violent presented increased folding patterns (gyrification) in the lateral OFC (right) compared to controls Absence of differences in OFC between groups
Yang et al., 2010	SZ with LHA such as murder (*n* = 22) SZ without LHA (*n* = 19) Murderers (*n* = 18) Healthy controls (*n* = 33)	34.68 ± 13.0 33.11 ± 10.09 31.39 ± 12.89 32.03 ± 9.89	14% ♂ 11% ♂ 16% ♂ 13% ♂	7.32 ± 4.22 11.63 ± 2.97 9.00 ± 5.45 8.83 ± 4.26	MRI (gray matter volume), 1.5 T	-	ROI: SFG, MFG, IFG, mOFG, lOFG), gyrus rectus (GR), parahippocampal gyrus (PHG), and hippocampus Covariates: IQ, anti-psychotic medications, years of education and whole brain volume	Absence of differences GMV PFC
**Functional**								
Wong et al., 1997	SZ with LHA (e.g., committed a homicide, disturbing behaviour unmanageable in a general hospital, etc.) (*n* = 17) SZ without LHA (*n* = 14) Healthy controls (*n* = 6)	36.2 ± 6.8 40.4 ± 10.1 35.2 ± 9.6	85% right 95% right-handed -	-	PET,	Resting	ROI: left (L) and right (R) anterior inferior temporal cortex (AIT), L and R anterior lateral temporal cortex (ALT), L and R anterior medial temporal cortex (AMT), L and R posterior inferior temporal cortex (PIT), L and R posterior lateral temporal cortex (PLT), L and R posterior medial temporal cortex (PMT), L and R temporal pole (TP), L and R inferior dorsolateral frontal cortex (IDLF), L and R superior dorsolateral frontal cortex (SDLF) and L and R orbito-medial frontal cortex (OMF) Covariates: - Groups matched for age and gender	Absence of group differences in FDG uptake in PFC regions
Fortier et al., 2023	SZ with LHA (e.g., stabbing, shooting, biting, assault, etc.) (*n* = 44) Healthy controls (*n* = 22)	34.30 ± 1.44 31.86 ± 1.79	100% ♂	92% right-handed 95% right-handed	fMRI, 3 T	Emotional Go/NoGo task (actors portraying anger (Angry) or no emotion (Neutral)	ROI: DLPFC, MFG, OFC, dACC and anterior insula Covariates: - Groups matched for age and handedness	In SZ (emotional inhibition contrast), lower connectivity between the left MFG (part of DMPFC and DLPFC) and the right OFC, as well as between the right MFG (part of DMPFC and DLPFC) and the right superior temporal gyrus compared to controls
Joyal et al., 2007	SZ with LHA (e.g., homicide, unplanned altercation with a friend under drug intoxication) + ASP + drugs (*n* = 12) SZ without LHA (*n* = 12) Healthy controls (*n* = 12)	27 ± 5 30 ± 9 28 ± 8	100% ♂	100% right-handed	fMRI, -	Computerized go/no-go	ROI: whole brain analysis and, posteriorly, prefrontal regions of interest BA (Brodmann Areas) 9, 10, 11, 44, 45, 46, and 47 Covariates: - Groups matched for age and handedness	SZ violent < OFC activation than controls and SZ non-violent
Kumari et al., 2006 (overlapped Barkataki et al., 2006)	SZ with LHA (e.g., fatal or near fatal act of violence against others) (*n* = 13) SZ without LHA (*n* = 12) Healthy controls (*n* = 13)	34.00 ± 4.86 33.85 ± 7.57 33.31 ± 6.85	100% ♂	100% right-handed	fMRI, 1.5 T	N-back-task	ROI: whole brain analysis Covariates: - Groups matched for age and IQ	Absence of SZ violent and healthy controls differences in DLPFC
Schiffer et al., 2017	ASPD SZ with LHA (e.g., violent acts against others) (*n* = 13) SZ with LHA (*n* = 16) Healthy violent (*n* = 18) SZ without LHA (*n* = 18) Healthy controls (*n* = 18)	34.4 ± 5.7 38.4 ± 9.0 35.3 ± 9.3 37.8 ± 8.3 36.3 ± 9.8	100% ♂	9.2 ± 0.4 9.4 ± 1.4 9.3 ± 0.8 9.8 ± 1.7 9.9 ± 1.1	fMRI, 1.5 T	Eyes test	ROI: whole brain analysis Covariates: - Groups matched for age, educational level, and IQ	Violent ASPD SZ higher activity in the medial PFC and left vlPFC than controls Violent SZ without ASPD in comparison with controls, absence differences
Tikàsz et al., 2018	SZ with LHA (e.g., violent attacks against others) (*n* = 24) SZ without LHA (*n* = 23) Healthy controls (*n* = 22)	36.0 ± 2.1 32.8 ± 1.8 31.9 ± 1.8	86% right 94% right 96% right 100% ♂	-	fMRI, 3 T	Affective Go/NoGo task (Ekman faces)	ROI: whole brain analysis and, posteriorly, the clusters that initially differed from groups were employed as data-driven ROI Covariates: positive symptoms (SZ)	Decreased activation in SZ violent relative to healthy men (right DLPFC)
**Intermittent explosive disorder**								
**Structural**								
Coccaro et al., 2016	IED with LHA (e.g., violent attacks against others) (*n* = 57) Psychiatric patients (*n* = 58) Healthy controls (*n* = 53)	34.4 ± 8.6 30.8 ± 9.0 31.2 ± 7.5	93% ♂ 83% ♂ 89% ♂	14.8 ± 2.0 16.2 ± 2.5 16.2 ± 2.0	MRI (gray matter volume), 3 T	-	ROI: whole brain analysis Covariates: age and gender	IED presented lower GMV in medial PFC and OFC (non-specified hemisphere) in comparison with controls and psychiatric patients → non-significant after controlling covariates
Rosell et al., 2010	IED with LHA (e.g., episodes of physical assault against other people) (*n* = 14) IED without LHA (*n* = 15) Healthy controls (*n* = 25)	36.75 ± 10.31 36.40 ± 9.25 32.86 ± 10.52	71.4% ♂ 80.0% ♂ 60.0% ♂	-	MRI (gray matter volume) and PET, -	-	ROI: OFC, OFC, DLPFC and MPFC; amygdala and parietal cortex Covariates: injected mass	Absence of volume differences in OFC, DLPFC and MPFC; and amygdala
Seok & Cheong, 2020	IED with LHA (e.g., violent attacks against others) (*n* = 15) Healthy controls (*n* = 15)	28.53 ± 2.36 28.60 ± 4.40	100% ♂ 100% right-handed	-	MRI (gray matter volume) and fMRI, 7 T	-	ROI for MRI: whole brain analysis Covariates: age, education, depressive symptoms, anxiety symptoms, impulsivity score, and intracranial volume	IED had significantly reduced GMV in left OFC relative to controls
**Functional**								
**Resting**								
Gan et al., 2019 (overlapped Gan et al., 2016)	IED with high anger expression-out (*n* = 11) Healthy controls (*n* = 12)	35.36 ± 7.4 30.7 ± 5.2	100% ♂ 100% ♂	13.5 ± 1.7 13.9 ± 1.4	fMRI, 4 T	rsFC	ROI: whole brain analysis Covariates: - Groups matched for age, educational level and ethnicity	Reactive aggressive individuals showed higher global efficiency in the left habenula, the left thalamus, the left DLPFC Reactive aggressive individuals showed lower clustering coefficient in the left precuneus, the left DMPFC, and left occipital regions relative to the control group
Rosell et al., 2010	IEI with LHA (e.g., physically aggressive) (*n* = 14) IEI without LHA (*n* = 15) Healthy controls (*n* = 25)	36.75 ± 10.31 36.40 ± 9.25 32.86 ± 10.52	71.4% ♂ 80.0% ♂ 60.0% ♂	-		PET	ROI: OFC, OFC, DLPFC and MPFC; amygdala and parietal cortex Covariates: injected mass	OFC 5-HT2AR availability was greater in IEI violence compared to patients without current physical aggression and healthy controls No significant differences in 5-HT2AR availability were observed in other brain regions examined
Volkow et al., 1995	IED with LHA (e.g., violent attacks against others without purposelessness) (*n* = 8) Healthy controls (*n* = 8)	34 ± 11 32 ± 7	100% ♂ 100% right-handed	-	PET	-	Quantitate glucose and lSF-deoxyglucose (FDG) Concentrations Covariates: - Groups matched for age and handedness	IED patients had significantly lower metabolism for left and right PFC
**Task**								
Coccaro et al., 2007	IED with LHA (LHA (e.g., violent attacks against others) (*n* = 10) Healthy controls (*n* = 10)	34.3 ± 7.3 30.9 ± 5.6	50% ♂ 50% ♂	15.6 ± 1.3 13.4 ± 1.0	fMRI, 3 T	Emotional facial expressions	ROI: whole brain analysis Covariates: - Groups matched for age, ethnicity, gender and socioeconomic status	IED presented lower left OFC activation than controls to happy faces IED presented lower left amygdala connectivity with the medial OFC (bilateral) than controls (negative correlation) in the task without differentiating the emotion. No positive correlations between amygdala-OFC signal were detected in HC subjects. In contrast, no correlations (positive or negative) between amygdala and OFC signal were observed in IED subjects
Gan et al., 2016	IED with high anger expression-out (*n* = 9) Healthy controls (*n* = 9)	34.4 ± 7.5 31.8 ± 6.5	100% ♂ 100% ♂	12.9 ± 1.4 13.7 ± 1.3	fMRI, 4 T	PSAP	ROI: whole brain analysis Covariates: - Groups matched for age, ethnicity, educational level and substance use	IED individuals exhibited lower bilateral VMPFC activation than controls
McCloskey et al., 2016	IED with LHA (e.g., violent attacks against others) (*n* = 20) Healthy controls (*n* = 20)	33.2 years 32.8 years	60% ♂ 60% ♂ 100% Right-handed	15.0 ± 1.7 15.9 ± 1.9	fMRI, 3 T	Angry (or Happy) face vs. Neutral face (emotional faces)	ROI: amygdala and ventral medial prefrontal/ OFC Covariates: - Groups matched for age, gender, ethnicity, and educational level	Absence of group differences in orbital medial PFC activation to angry (or happy) faces During angry facial processing, IED presented positive amygdala-OMPFC coupling, and controls exhibited significant negative amygdala-OMPFC coupling
Moeller et al., 2014	IED with LHA with high anger expression-out (*n* = 11) Cocaine without LHA (*n* = 21) Healthy controls (*n* = 17)	33.5 ± 7.1 43.2 ± 6.5 32.6 ± 6.4	100% ♂ 100% Right-handed	13.5 ± 1.7 13.0 ± 2.0 13.5 ± 1.4	fMRI, 4 T	Color-word Stroop task	ROI: whole brain analysis Covariates: age and cigarette smoking history	IED higher activation than controls in superior frontal gyrus (DLPFC) and lower than controls in middle frontal gyrus (DLPFC)
Seok & Cheong, 2020	IED with LHA (e.g., violent attacks against others) (*n* = 15) Healthy controls (*n* = 15)	28.53 ± 2.36 28.60 ± 4.40	100% ♂ 100% right-handed	-	MRI (gray matter volume) and fMRI, 7 T	Anger processing (videos)	ROI for fMRI: bilateral putamen, bilateral insula, right amygdala, right anterior cingulate cortex and bilateral middle/inferior frontal gyri (Brodmann area [BA] 9) Covariates: depressive symptoms, anxiety symptoms, and impulsivity score	Absence of differences in activation of bilateral middle/inferior frontal gyri (part of VLPFC) during anger processing
**Other mental disorders**								
Bertsch et al., 2018	BPD anger-prone (*n* = 30) Healthy controls (*n* = 28)	26.9 ± 6.1 26.5 ± 5.7	100% ♀	-	fMRI	Approach-avoidance task (faces)	ROI: left and right lateral anterior PFC (aPFC) and DLPFC Covariates: - Groups matched for age and IQ	BPD presented lower activation in left dlPFC than controls after establishing differences between incongruent and congruent trials. Nonetheless, it was the opposite in right dlPFC (BPD > controls). Regarding connectivity during incongruent – congruent trials, left dlPFC negatively associated with right amygdala in controls, but there was absent in BPD
Bertsch et al., 2019	BPD anger-prone (*n* = 15) Healthy controls (*n* = 25)	28.3 ± 8.9 30.1 ± 5.9	100% ♂	-	fMRI	Approach-avoidance task (faces)	ROI: left and right aPFC and dlPFC. Coupling of left and right aPFC and DLPFC with the amygdala Covariates: - Groups matched for age and IQ	Male patients with BPD showed reduced anterolateral prefrontal activations during emotional action control compared to controls Neither the connectivity analyses with the left and right aPFC as seed regions nor additional connectivity analyses with the left and right dlPFC as seeds revealed any significant coupling with the amygdala
Herpertz et al., 2017	BPD anger-prone men (*n* = 23) BPD anger-prone female (*n* = 33) Healthy control men (*n* = 26) Healthy control female (30)	30.65 ± 8.75 26.19 ± 5.70 31.09 ± 6.65 27.69 ± 6.38	-	10.87 ± 1.36 11.64 ± 1.46 11.72 ± 1.43 12.21 ± 1.19	fMRI	Script-driven-imagery paradigm (audio)	ROI: amygdala, ACC, OFC, and DLPFC Covariates: age, IQ, and years of education	BPD men presented higher activation in the right DLPFC during aggression phase than control men Absence of group differences in the case of women
Dougherty et al., 2004	Depressive with violent attacks (*n* = 10) Depressive without violent attacks (*n* = 10) Healthy controls (*n* = 10)	35.40 ± 14.62 36.90 ± 9.33 33.90 ± 11.85	-	100% right-handed	PET	Describing life angry events	ROI: VMPFC and amygdala Covariates: - Groups matched for age, gender and severity of depression	Depressive violent individuals presented lower activation in the left VMPFC during the anger vs neutral comparison than controls During anger induction in the depressive violent individuals there was a positive correlation between the left VMPFC and the left amygdala. There was a significant inverse correlation between left VMPFC with the left amygdala in controls
Chang et al., 2017	Conduct disorder (*n* = 14) Healthy controls without control disorder (*n* = 8)	26.71 ± 4.32 29.38 ± 2.83	100% ♂	-	PET	Resting	ROI: midbrain, striatum, thalamus, PFC, and the cerebellum Covariates: - Groups matched for age	Absence of differences between groups in PFC (serotonin reuptake transporter)
Lanctôt et al., 2004	Violent patients with Alzheimer (*n* = 30) Non-violent patients with Alzheimer (*n* = 19)	74.0 ± 10.7	51% ♂	-	SPECT	Resting	ROI: OFC, middle and inferior regions of medial temporal gyrus, anterior cingulate corte and hypothalamus) Covariates: age, sex and MMSE score	Absence of differences in regional cerebral blood flow in OFC

*Note*: *ACC* anterior cingulate cortex, *aPFC* anterior prefrontal cortex, *BDP* borderline personality disorder, *dACC* dorsal anterior cingulate cortex, *DLPFC* dorsolateral prefrontal cortex, *DMPFC* dorsomedial prefrontal cortex, *FDG* F-fluorodeoxyglucose, *fMRI* functional magnetic resonance imaging, *IED* intermittent explosive disorder, *GMD* gray matter density, *GMV* gray matter volume, *LHA* life history of aggression, *MMSE* Mini-Mental State Examination, *MFG* medial frontal gyrus, *MRI* magnetic resonance imaging, *MTG* middle temporal gyrus, *OFC* orbitofrontal cortex, *PET* positron emission tomography, *SMA* supplementary motor area, *PFC* prefrontal cortex, *SZ* schizophrenia, *VLPFC* ventrolateral prefrontal cortex, *VMPFC* ventromedial prefrontal cortex

#### Intermittent explosive disorder (IED)

A total of ten empirical studies were included in this section, with three of them addressing the structural section (Coccaro et al., 2016; Rosell et al., 2010; Seok & Cheong, 2020) and the other seven the functional analysis (Coccaro et al., 2007; Gan et al., 2016, 2019; McCloskey et al., 2016; Moeller et al., 2014; Rosell et al., 2010; Volkow et al., 1995). Most studies were conducted with men (Gan et al., 2016, 2019; Moeller et al., 2014; Seok & Cheong, 2020; Volkow et al., 1995), except for several studies which combined men and women (Coccaro et al., 2007, 2016; Moeller et al., 2014; Rosell et al., 2010).

According to the findings of structural studies, two of the three studies (67%) included MRI and reported that IED patients showed lower GMV in the medial PFC and OFC compared to controls (Coccaro et al., 2016; Seok & Cheong, 2020). Specifically, one of the studies indicated that differences between groups in OFC were exclusively located on the left OFC (Seok & Cheong, 2020).

Structural conclusions could be complemented by results from functional studies that measured PFC activation or functioning during a resting period. First, it was established that IEI patients exhibited higher 5-HT2A receptor availability in the OFC compared to controls (Rosell et al., 2010), as well as lower glucose metabolism in the PFC (bilaterally) (Volkow et al., 1995). Furthermore, IEI patients characterized by high anger expression-out showed higher rsFC between the left habenula, left thalamus, and left DLPFC compared to controls. However, they also demonstrated lower rsFC between the left precuneus, left DMPFC, and left occipital regions compared to controls (Gan et al., 2019).

Regarding PFC activation or functional connectivity in response to laboratory tasks, five studies analyzed group differences (Coccaro et al., 2007; Gan et al., 2016; McCloskey et al., 2016; Moeller et al., 2014; Seok & Cheong, 2020). Three of them measured whether differences exist between groups processing emotional facial stimuli or emotional videos (Coccaro et al., 2007; McCloskey et al., 2016; Seok & Cheong, 2020) and the others assessed whether groups differed in anger or frustration tasks (Gan et al., 2016; Moeller et al., 2014). Only one of these studies failed to report significant group differences (Seok & Cheong, 2020). Particularly, it seemed that IED patients with a history of several episodes of violent attacks against others presented lower left OFC activation than controls when processing happy faces (Coccaro et al., 2007). Moreover, when researchers did not differentiate the emotional valence of faces, IEI patients exhibited lower connectivity between the left amygdala and the medial OFC (bilateral) in comparison with controls (Coccaro et al., 2007). Additionally, IED participants exhibited positive amygdala-OMPFC coupling with angry facial expressions, with controls showing the opposite pattern (McCloskey et al., 2016). Tasks that measured aggressive behavior (e.g., PSAP) pointed out that IED individuals exhibited lower bilateral VMPFC activation compared to controls (Gan et al., 2016). Furthermore, the employment of a task measuring the inhibitory control (the Stroop test) revealed that IED patients showed higher activation than controls in the superior frontal gyrus (part of the DLPFC) and lower activation than controls in the middle frontal gyrus (part of the DLPFC) (Moeller et al., 2014) (Table 2).

#### Other mental disorders (borderline personality disorder, depression, conduct disorder Alzheimer, or non-defined)

A total of four studies were included in this section (Bertsch et al., 2018, 2019; Dougherty et al., 2004; Herpertz et al., 2017). Three of them involved patients with borderline personality disorder (BPD) (Bertsch et al., 2018, 2019; Herpertz et al., 2017), while the other focused on patients with depression and violence (Dougherty et al., 2004). Although the studies involving BPD patients included participants of both sexes, the study with depression patients did not provide information on gender distribution.

Regarding BPD patients who were anger-prone, women affected by BPD exhibited lower activation during an inhibiting emotional task in the left DLPFC and higher in the right DLPFC compared to healthy controls (Bertsch et al., 2018). Furthermore, men with BPD showed lower anterolateral PFC activations during this task compared to controls (Bertsch et al., 2019). The other study combined men and women affected by BPD. This study observed that in an anger induction task, only male patients showed higher activation in the right DLPFC during the aggression phase compared to control men (Herpertz et al., 2017).

Offenders with conduct disorders did not differ from controls in serotonin reuptake transporter in the PFC (Chang et al., 2017).

With regard to patients with Alzheimer’s disease, no differences in regional cerebral metabolic rate of glucose in the OFC were found between those defined as violent from those who did not present a life history of aggression (Lanctôt, et al., 2004). Regarding the analysis of violent and depressive patients, it could be concluded that in the anger induction task, depressive patients showed lower activation in the left VMPFC during the anger vs neutral task compared to controls. In fact, during the anger induction phase, the depressive violent patients exhibited a positive correlation between the left VMPFC and the left amygdala. Nevertheless, the control group showed the opposite pattern of connectivity between the left VMPFC and the left amygdala (Dougherty et al., 2004) (Table 2).

#### Offenders diagnosed with antisocial personality disorder (ASPD)

A significant portion of the studies in this section focused on conclusions drawn from research involving men. In total, seven articles were included, all of which used MRI techniques to assess group differences between men with ASPD who had committed violent crimes and control groups. It was found that 71% of the studies involving adults with ASPD showed differences in the PFC compared to controls (Bertsch et al., 2013; Laakso et al., 2002; Narayan et al., 2007; Raine et al., 2000, 2011), while the remaining studies did not find significant group differences (Barkataki et al., 2006; Gregory et al., 2012). A key study indicated that individuals with ASPD had lower GMV in the total PFC compared to controls, with significant differences noted in the right hemisphere (Raine et al., 2000). Further research specified that the reduced GMV in individuals with ASPD was more pronounced in the OFC and parts of the DLPFC compared to controls (Laakso et al., 2002; Raine et al., 2011). These findings align with those from a study involving ASPD offenders with borderline personality disorder, which found lower GMV in the left OFC and DLPFC, as well as in the right VMPFC compared to controls (Bertsch et al., 2013). Additionally, individuals with ASPD showed reduced cortical thickness in the inferior medial frontal cortices on both sides, which are part of the lateral PFC, when compared to controls (Narayan et al., 2007). Notably, the study by Raine et al., (2012) examined white-collar criminals who displayed increased cortical thickness in the left VMPFC compared to controls, indicating a contrasting difference from those with ASPD.

The analysis of PFC functioning during resting conditions was assessed in four studies (Basoglu et al., 2008; Goyer et al., 1994; Kolla et al., 2015, 2021). From those studies, three failed to report significant differences between groups and only one of them reported differences (25%). In fact, it seemed that ASPD individuals did not differ from controls in the right DLPFC ratio of N-acetyl aspartate (NAA), creatine (Cr) and choline-related compounds (Basoglu et al., 2008). It was also found that groups did not differ in levels of OFC fatty acid amide hydrolase (Kolla et al., 2021) or glucose metabolism in this PFC region (Goyer et al., 1994). Nevertheless, ASPD individuals showed reduced MAO-A distribution volumes, an index of MAO-A density, in the OFC, DLPFC, VLPFC and MPFC, when compared to controls (Kolla et al., 2015).

Regarding the functional analysis, four studies assessed the differences between groups when individuals were submitted to different tasks (Gregory et al., 2015; Kumari et al., 2006; Schiffer et al., 2017; Schiffer et al., 2014). Two of them failed to report group differences (Gregory et al., 2015; Kumari et al., 2006), the other concluded that ASPD offenders exhibited significantly lower activity in the left DLPFC compared to controls (Schiffer et al., 2014) (Table 3).

**Table 3:** Summary of characteristics of the studies assessing group differences in prefrontal cortex in individuals with personality disorders and history of violent behaviors, alphabetically ordered by first author’s surname

Authors	Sample	Age	Gender	Educational level	Neuroimaging characteristics	Laboratory task	ROI	Results
**Antisocial personality disorder**								
**Structural**								
Barkataki et al., 2006	ASPD offenders (*n* = 13) Healthy controls (*n* = 15)	31.62 ± 8.03 32.13 ± 7.47	100% ♂	-	MRI (gray matter volume), 1.5 T	-	ROI: whole brain analysis Covariates: whole brain volume	Absence of group differences in PFC
Bertsch et al., 2013	ASPD with BPD offenders (*n* = 13) Non-offender controls (*n* = 14)	28.9 ± 10.1 26.1 ± 8.3	100% ♂	-	MRI (gray matter volume), 1.5 T	-	ROI: frontal pole, OFC, VMPFC, DMPFC, DLPFC, inferior frontal cortex (IFC), insula (INS), hippocampal-amygdalar formation (HAF), lateral temporal lobe (LTL), temporal pole (TP), posterior cingulate/precuneus (PCC/PC), postcentral gyrus (PCG), and occipital cortex Covariates: total intracranial volume	ASPD-BPD offenders lower GMV in left OFC and right VMPFC than controls. Moreover, APD-BPD offenders also presented lower GMV in left DLPFC than controls
Gregory et al., 2012	ASPD + psychopathy offenders (*n* = 17) ASPD offenders (*n* = 27) Non-offender controls (*n* = 22)	38.9 ± 9.4 36.1 ± 8.2 32.4 ± 7.7	100% ♂	-	MRI (gray matter volume), 1.5 T	-	ROI: anterior rostral PFC (6 subregions), temporal poles (6 subregions) and anterior insula (6 subregions) Covariates: age and IQ	Offenders with antisocial and psychopathy presented reduced GMV in the anterior rostral prefrontal cortex (bilaterally) than offenders without psychopathy and controls Offenders with ASPD without psychopathy did not differ from controls
Laakso et al., 2002	ASPD + alcohol offenders (*n* = 24) Healthy controls (*n* = 33)	34 ± 10 31 ± 8	100% ♂	9 ± 1 14 ± 2	MRI (gray matter volume), 1.5 T	-	ROI: superior, middle and inferior frontal gyri, and covered Brodmann areas 8, 9, 10, and 46 and part of area 45 Covariates: education and duration of alcoholism	ASPD + alcohol lower GMV in left DLPFC and left OFC than controls Nonetheless, absence of differences in DLPFC and OFC right
Narayan et al., 2007	ASPD with LHA (e.g., frequent and severe violent attacks against others) (*n* = 14) Healthy controls (*n* = 15)	33.5 ± 10.4 32.1 ± 7.5	100% ♂ 100% Right-handed	-	MRI (gray matter thickness), 1.5-T	-	ROI: whole brain analysis Covariates: age and total brain volume	ASPD group have lower cortical thickness in (inferior medial frontal cortices bilaterally, which is part of lateral PFC) than controls
Raine et al., 2000	ASPD (*n* = 21) Substance misuse (*n* = 26) Healthy controls (*n* = 34)	31.9 ± 6.8 30.2 ± 6.2 30.4 ± 6.7	100% ♂	35.4 ± 9.4 32.6 ± 11.4 34.1 ± 10.0 Majorly right-handed	MRI (gray matter volume), 1.5 T	-	ROI: whole brain analysis Covariate: whole brain volume	ASPD lower GMV in PFC (total) than controls (11%) and substance groups (13.9%) Increased right relative to left PFC GMV
Raine et al., 2011	ASPD (*n* = 18) Substance misuse (*n* = 24) Healthy controls (*n* = 30)	From 21 to 45	100% ♂	-	MRI (gray matter volume), 1.5 T	-	ROI: PFC (superior frontal, middle frontal, inferior frontal, orbito-frontal, and rectal gyri) Groups matched for whole brain volume	ASPD males compared to male controls showed an 8.7% reduction in GMV of OFC, a 17.3% reduction in middle frontal gyrus (part of DLPFC) than controls and substance group APDs had significantly reduced right middle frontal volumes compared to controls and substance groups. APDs also showed significantly reduced left middle frontal volumes compared to both groups of controls
**White collar criminals**								
Raine et al., 2012	White collar criminals (*n* = 21) Controls matched for blue collar crimes (*n* = 21)	30.38 ± 7.95 29.38 ± 6.73	86% ♂ 86% ♂	- Majorly right-handed	MRI, 1.5 T	-	ROI: whole brain analysis Covariates: - Groups matched for age, gender, ethnicity, and general level of criminal offending (blue collar crimes)	White collar criminals showed increased cortical thickness in the left VMPFC, right inferior frontal gyrus, the right precentral gyrus and the right postcentral than controls
**Functional**								
**Resting**								
Basoglu et al., 2008	ASPD with LHA (e.g., convicted with violent crimes that such as first-degree murder, manslaughter and assault with physical injury to others) (*n* = 15) Healthy controls (*n* = 15)	25.4 ± 7.2 25.1 ± 7.2	100% ♂ 100% right-handed	7.2 ± 2.3 7.2 ± 7.7	MRS, 1.5 T	-	ROI: right DLPFC, ACC, amygdala and hippocampus Covariates: - Groups matched for age and educational level	Absence of differences in right DLPFC ratio of N-acetyl aspartate (NAA), creatine (Cr) and choline-related compounds
Goyer et al., 1994	ASP with LHA (e.g., Aggressive behavior directed toward others) (*n* = 6) Healthy controls (*n* = 43)	23.0 ± 3.3 30.2 ± 9.2	100% ♂ 49% ♂	-	PET (metabolic rates of glucose)	-	ROI: frontal cortex Covariates: age and gender	Absence of group differences in metabolic glucose rate in anterior OFC
Kolla et al., 2015	ASPD offenders (*n* = 18) Non-offender controls (*n* = 18)	36.2 ± 9.4 36.4 ± 8.9	100% ♂	100% right-handed	PET, 1.5-T	-	ROI: PFC Covariates: - Groups matched for age, gender, alcohol dependence and absence of psychotropic medication use	The main finding is that MAO-A VT was significantly lower in ASPD versus controls, specifically, in OFC, DLPFC, VLPFC and MPFC
Kolla et al., 2021	ASPD offenders (*n* = 16) Non-offender controls (*n* = 16)	35.6 ± 9.4 30.0 ± 9.5	100% ♂ 31% with SZ with each group	13.0 ± 2.3 16.0 ± 2.2	PET, 3 T	-	ROI: whole brain analysis Covariates: educational level	Absence of fatty acid amide hydrolase levels in OFC between groups Absence significant correlations between fatty acid amide hydrolase in OFC with aggression
**Laboratory task**								
Gregory et al., 2015	ASPD + psychopathy offenders (*n* = 18) ASPD offenders (*n* = 20) Non-offender controls (*n* = 18)	40.1 ± 8.9 36.8 ± 7.6 34.8 ± 8.8	100% ♂	-	fMRI, 1.5 T	Probabilistic response reversal task (punishment vs reward)	ROI: whole brain analysis Covariates: - Groups matched for age and IQ	Absence of group differences in PFC activation to task
Kumari et al., 2009 (overlapped Barkataki et al., 2006)	ASPD SZ with LHA (e.g., serious violent attacks against others) (*n* = 10) Healthy controls (*n* = 13)	31.30 ± 8.14 33.31 ± 6.85	100% ♂	100% right-handed	fMRI, 1.5 T	N-back-task	ROI: whole brain analysis Covariates: - Groups matched for age and IQ	Absence differences in DLPFC of SZ violent and healthy controls
Schiffer et al., 2014	ASPD offenders (*n* = 21) Healthy controls (*n* = 23)	35.2 ± 8.2 34.1 ± 8.9	100% ♂ 100% right-handed	9.56 ± 1.12 9.76 ± 1.09	fMRI, 1.5 T	Stroop task	ROI: ACC and dlPFC Covariates: three impulsivity measures and PCL factor 1	Offenders with ASPD,relative to non-offenders exhibited significantly lower activation in the left DLPFC
**Psychopathy**								
**Structural**								
Bertsch et al., 2013	ASPD with psychopathy offenders (*n* = 12) Non-offender controls (*n* = 14)	28.9 ± 10.1 27.3 ± 5.4 26.1 ± 8.3	100% ♂	-	MRI (gray matter volume), 1.5 T	-	ROI: frontal pole, OFC, VMPFC, DMPFC, DLPFC, inferior frontal cortex (IFC), insula (INS), hippocampal-amygdalar formation (HAF), lateral temporal lobe (LTL), temporal pole (TP), posterior cingulate/precuneus (PCC/PC), postcentral gyrus (PCG), and occipital cortex Covariates: total intracranial volume	APD psychopathy presented lower GMV in DMPFC (bilaterally) than controls
Boccardi et al., 2011 (overlapped Tiihonen et al., 2008)	Violent psychopath offenders (*n* = 26) Non-offender controls (*n* = 25)	32.5 ± 8.4 34.6 ± 10.8	100% ♂	-	MRI (gray matter density), 1.0 T	-	ROI: cerebral cortex and amygdala Covariates: PCL scores	OFC (left and right): Offenders < controls
Kolla et al., 2014 (overlapped Gregory et al., 2012)	Psychopathy offenders (*n* = 9) Offenders (*n* = 15) Non-offender controls (*n* = 13)	38.7 ± 6.0 35.0 ± 9.3 35.1 ± 8.0	100% ♂	-	MRI (gray matter volume), 1.5 T	-	ROI: whole brain analysis Covariates: - Groups matched for age, IQ and substance misuse	Absence of PFC differences
Kolla et al., 2017	Psychopathy offenders (*n* = 18) Non-offender controls (*n* = 20)	35 years	100% ♂	100% right-handed	MRI (volume and cortical thickness), 3 T	-	ROI: amygdala, middle and lateral orbitofrontal cortex Covariates: - Groups matched for age, IQ and ethnicity	Psychopath group presented decreased cortical thickness in the left lateral OFC gyrus compared with controls. There was no group effect for cortical thickness in the right middle OFC gyrus or the right lateral OFC gyrus
**Functional**								
Birbaumer et al., 2005	Psychopathy offenders (*n* = 10) Non-offender controls (*n* = 10)	35.30 ± 5.79 31.50 ± 7.58	100% ♂	12.80 ± 1.69 12.60 ± 2.37	fMRI, 1.5 T	Aversive differential delay conditioning	ROI: left amygdala, left anteromedial OFC, anterior and posterior cingulate, right anterior and left middle insula, supplementary motor area, and secondary somatosensory cortex bilaterally Covariates: - Groups matched for age and educational level	Psychopaths lack OFC activation, especially in the second half of the acquisition phase when the learned association needs to be translated into behavioral responding
Geurts et al., 2016	Psychopathy offenders (*n* = 14) Non-offenders without APD (*n* = 10) Non-offenders with APD (*n* = 10)	40.1 ± 8.8 42.5 ± 10.22 39.1 ± 9.7	100% ♂	79% right-handed 15% right-handed (for both groups of controls)	fMRI, 1.5 T	Monetary incentive delay task	ROI: Connectivity ventral striatum with other brain structures (reward expectation) Covariates: - Groups matched for age and IQ	Psychopathic higher connectivity between ventral striatum and DMPFC than controls
Kiehl et al., 2004	Psychopathy offenders (*n* = 8) Non-offender controls (*n* = 8)	33.9 ± 7.6 27.9 ± 5.0	100% ♂	9.9 ± 3.5 12.4 ± 0.74 100% right-handed	fMRI, 1.5 T	Concrete/abstract lexical decision task	ROI: inferior frontal gyrus, cingulate gyrus, middle frontal gyrus, insula, inferior and superior parietal lobule, superior and medial temporal gyrus, fusiform gyrus and thalamus (bilateral) Covariates: - Groups matched for age, educational level, IQ, socioeconomic status and handedness	Absence of group differences in inferior and middle frontal gyrus (part of VLPFC and DLPFC, respectively)
Müller et al., 2003	Psychopathy offenders (*n* = 6) Non-offender controls (*n* = 6)	33.0 ± 8.0 28.0 ± 4.14	100% ♂	100% right-handed	fMRI, 1.5 T	International Affective Picture System	ROI: Frontal lobe gyrus, paraecentralis, gyrus, frontalis inferior, gyrus paraecentralis, temporal lobe, gyrus temporalis superior, gyrus temporalis medius, gyrus temporalis medius, parietal lobe, gyrus supramarginalis, gyrus angularis, occipital lobe, gyrus fusiformis, gyrus fusiformis and cerebellum (bilaterally) Covariates: - Groups matched for age and handedness	Psychopaths presented higher activation in inferior frontal gyrus (bilateral) than controls in emotional stimulus
Schneider et al., 2000	Psychopathy offenders (*n* = 12) Non-offender controls (*n* = 12)	31.50 ± 8.21 27.58 ± 4.56	100% ♂	100% right-handed	fMRI, 1.5 T	Aversive classical conditioning odors	ROI: amygdala, thalamus, cingulate gyrus anterior and posterior, OFC, dlPFC, temporal superior, medial and inferior cortex, occipital cortex, precuneus, and cerebellum Covariates: - Groups matched for age and handedness	Higher activation in DLPFC during acquisition period
Volman et al., 2016	Psychopathic offenders (*n* = 15) Non-offender controls (*n* = 19)	37.8 ± 7.9 40.7 ± 10.3	100% ♂ 81% 62% right-handed	-	fMRI, 3 T	Approach-avoid emotional faces	ROI: aPFC Covariates: - Groups matched for age, IQ and handedness	Psychopathic offenders show reduced aPFC activity as well as less aPFC–amygdala connectivity during the control of emotional behavior

*Note*: *aPFC* anterior prefrontal cortex, *ASPD* antisocial personality disorder, *dACC* dorsal anterior cingulate cortex, *DLPFC* dorsolateral prefrontal cortex, *DMPFC* dorsomedial prefrontal cortex, *FDG* F-fluorodeoxyglucose, *fMRI* functional magnetic resonance imaging, *GMD* gray matter density, *GMV* gray matter volume, *LHA* life history of aggression, *MRI* magnetic resonance imaging, *MTG* middle temporal gyrus, *OFC* orbitofrontal cortex, *PCL* Psychopathy Checklist, *PET* positron emission tomography, *SMA* supplementary motor area, *PFC* prefrontal cortex, *VLPFC* ventrolateral prefrontal cortex, *VMPFC* ventromedial prefrontal cortex

#### Offenders diagnosed with psychopathy who perpetrated violent crimes

This section included a total of ten studies mainly based on men, except for one that contained a small percentage of women (Volman et al., 2016). Regarding the analysis of PFC structural differences between offenders diagnosed with psychopathy who have committed violent crimes and controls, a total of four studies were included in this section (Bertsch et al., 2013; Boccardi et al., 2011; Kolla et al., 2014, 2017). From those studies, only Kolla et al., (2014) failed to report group differences. Therefore, 75% of the studies highlighted the existence of PFC structural differences between the above-mentioned groups. In this sense, two of them found that individuals diagnosed with psychopathy exhibited lower GMV in the OFC (bilaterally) (Boccardi et al., 2011) as well as lower cortical thickness in the left lateral OFC gyrus compared to controls (Kolla et al., 2017). The third study found that psychopathic individuals showed lower GMV in the DMPFC (bilaterally) compared to controls (Bertsch et al., 2013).

Studies measuring PFC activation during a resting period were not included. Therefore, the measurement of PFC activation during specific tasks included a total of six studies (Birbaumer et al., 2005; Geurts et al., 2016; Kiehl et al., 2004; Müller et al., 2003; Schneider et al., 2000; Volman et al., 2016). Only one of them failed to report group differences in the activation of the inferior and middle frontal gyrus (part of VLPFC and DLPFC, respectively) during a lexical decision task (Kiehl et al., 2004). Conversely, two studies employing a task for emotional processing concluded that psychopaths showed higher activation of the inferior frontal gyrus (bilateral), part of the VLPFC (Müller et al., 2003), as well as the lower anterior PFC when compared to controls (Volman et al., 2016). Moreover, it could be concluded that psychopaths did not exhibit OFC activation during the phase that establishes the association between stimulus and behavior in the aversive conditioning task (Birbaumer et al., 2005). However, they also showed higher activation in the DLPFC during this period when compared to controls (Schneider et al., 2000). Additionally, compared to controls psychopaths showed higher connectivity between the ventral striatum and DMPFC in a monetary incentive delay task (Geurts et al., 2016). This was complemented by another study which revealed that, compared to controls, psychopaths exhibited lower connectivity between the anterior PFC and the amygdala when required to control their emotions (Volman et al., 2016) (Table 3).

#### Correlation or regression analysis between PFC measurements and violence perpetrated against others

Specifically, a total of 24 studies were included in this section distributed as follows: 11 including structural analysis and the other 13 functioning, activation or functional connectivity between brain regions. The studies were primarily or entirely conducted with men, accounting for approximately 76% of the research. Furthermore, much of the empirical research included focused on individuals with an average age of 35 years old. Regarding the obtained results, from all the studies included, only 25% failed to find a significant association between PFC and violence, which was measured with self-reports and laboratory tasks or the combination of both (see Table 4). As described below, there were differences depending on the assessment of the structural or functional PFC regarding the violence measurement.

**Table 4:** Summary of correlation or regression analysis measuring the relationship between neuroimaging results with violence-related concepts in several violent samples, alphabetically ordered by first author’s surname

Authors	Sample	Age	Gender	Educational level	Neuroimaging characteristics	ROI and covariates		Results
**Structural**								
**Life history of aggression**								
Antonucci et al., 2006	Psychiatric patients with LHA (e.g., violent attacks against others) (*n *= 15)	39 ± 5.8	73% ♂	10.8 ± 2.1	MRI (gray matter volume), 1.5 T	ROI: OFC Controlling for whole brain volume		Affective disorder (*n* = 8) GMV right OFC with LHA (*r* = -.03, ns); GMV left OFC with LHA (*r* = -.53, ns); GMV total OFC with LHA (*r* = -.29, ns) Non-affective disorder (*n* = 7) GMV right OFC with LHA (*r* = -.05, ns); GMV left OFC (cc) with LHA (*r* = -.46, ns); GMV total OFC with LHA (*r* = -.35. ns)
Bounoua et al., 2022	Individuals with LHA (e.g., physical aggression against others) (*n* = 134)	From 18 to 55	-	-	MRI (cortical thickness), 3 T	ROI: whole cortical regions Partial correlations without specifying the covariated variable		Cortical thickness in the superior frontal gyrus (part of DMPFC and DLPFC) and the caudal medial frontal gyrus (part of the DMPFC and DLPFC) was negatively associated with LHA (*r* = -.29, *p* =.001 and *r* = -.18, *p* =.038)
Coccaro et al., 2018	Physically healthy, adult, same-sexed twins with LHA (e.g., violent attacks against others) (*n* = 287 twins)	35.0 ± 8.9	50.5% ♂	-	MRI (gray matter volume), 3 T	ROI: whole brain analysis -		GMV mPFC,with LHA (*n* = 41; MZ twin 1; *r* = −.24, p non-reported) GMV mPFC,with LHA (*n* = 41; MZ twin 2; *r* = −.23, p non-reported) GMV lPFC with LHA (*n* = 41; MZ twin 1; *r* = −.07, p non-reported) GMV lPFC with LHA (*n* = 41; MZ twin 2; *r* = −.29, p non-reported) GMV mPFC,with LHA (*n* = 27; DZ twin 1; *r* = −.17, p non-reported) GMV mPFC,with LHA (*n* = 27; DZ twin 2; *r* = −.15, p non-reported) GMV lPFC with LHA (*n* = 27; MZ twin 1; *r* = −.29, p non-reported) GMV lPFC with LHA (*n* = 27; DZ twin 2; *r* = −.25, p non-reported)
Drachman et al., 2022	BDP with LHA (e.g., aggression towards others or violent behaviour) (*n* = 38) Healthy controls (*n* = 29)	35.7 ± 11.5 38.3 ± 11.2	26% ♂ 36% ♂	-	MRI (gray matter volume), 3 T	ROI: whole brain analysis Controlling for total intracranial volume		In borderline patients, GMV in bilateral OFC correlated negatively with LHA (statistics non-provided)
Gansler et al., 2009	Psychiatric patients with LHA (e.g., verbal/physical aggression and consequences of anti-social behaviors) (*n* = 41) Controls (*n* = 19)	40.12 ± 8.3 40.94 ± 7.5	88% ♂; 76% right-hand 95% ♂ 84% right-hand	10.80 ± 2.9 15.53 ± 1.9	MRI (gray matter volume), 1.5 T	ROI: OFC Controlling for total intracranial volume, years of education, self-reported impulsivity and the volume of the opposite region		In psychiatric patients, GMV of left OFC and total OFC were associated with LHA (*r* = −.48, *p* <.01; *r* = −.59, *p* <.01, respectively), but this variable was unrelated to GMV of right OFC (*r* = −.23, *p* =.14) In controls, GMV of left and right OFC were unrelated to LHA (*r* = −.21, *p* =.38; *r* = −.00, *p* =.99, respectively). They did not calculate the correlation for the total OFC
Seok & Cheong, 2020	IED with LHA (e.g., violent attacks against others) (*n* = 15) Healthy controls (*n* = 15)	28.53 ± 2.36 28.60 ± 4.40	100% ♂ 100% right-handed	-	MRI (gray matter volume) and fMRI, 7 T	ROI for MRI: whole brain analysis ROI for fMRI: bilateral putamen, bilateral insula, right amygdala, right anterior cingulate cortex -		GMV of PFC, specifically, left OFC was unrelated to LHA in each group (statistics non-provided)
Gorka et al., 2018	IED with LHA (e.g., violent attacks against others) (*n* = 53)	34.0 ± 10.9	52.8% ♂	-	fMRI, 3 T	Cyberball is a virtual ball-tossing game ROI: PFC -		Less VLPFC activation was also associated with greater LHA (*b* = −4.09, SE = 1.81, *p* <.05) Greater prospective intolerance of uncertainty was associated with less VLPFC activation (a path: *b* = −0.05, Greater prospective-IU was associated with less left VLPFC activation during social exclusion
Hoptman et al., 2010	SZ with LHA (e.g., violent attacks against others) (*n* = 25) Healthy controls (*n* = 21)	36.7 ± 10.5 40.4 ± 10.8	88% ♂ 76% ♂	12.3 ± 2.1 15.5 ± 3.0	rsFC, 1.5 T	ROI: amygdala connectivity with cingulate, insula, DLPFC parahippocampal gyrus and OFC -		All sample, amygdala/vPFC with BPAQ scores (*n* = 45; *r* = -.55, *p* <.001 SZ, amygdala/vPFC with BPAQ scores; *r* = -.66, *n* = 24, *p* =.0004; left hemisphere *r* = -.44, *p* =.033; right *r* = -.56, *P* =.005) SZ, amygdala/vPFC with LHA total score (left *r* *=* -.24, *n* = 22, *p* =.29; right *r* = -.41, *p* =.057; bilateral *r* = -.46, *p* =.029) and total arrests (left *r* = -.34, *n* = 17, *p* =.18; right *r* = -.64, *p* =.005; bilateral *r* = -.65, *p* =.005) In controls, non-significant OFC or DLPFC non-significant associated with BPAQ
**Aggression**								
Antonucci et al., 2006	Psychiatric patients with LHA (e.g., violent attacks against others) (*n* = 15)	39 ± 5.8	73% ♂	10.8 ± 2.1	MRI (gray matter volume), 1.5 T	ROI: OFC Controlling for whole brain volume		Affective disorder (*n* = 8) GMV R OFC with AQ Physical Aggression (*r* = −.17, ns); GMV L OFC with AQ Physical Aggression (*r* = −.66, ns); GMV Total OFC with AQ Physical Aggression (*r* = −.44, ns) Non-affective disorder (*n* = 7) GMV R OFC with AQ Physical Aggression (*r* = −.60, ns); GMV L OFC with AQ Physical Aggression (*r* = −.54, ns); GMV Total OFC with AQ Physical Aggression (*r* = −.64, ns)
da Cunha-Bang et al., 2017a	Violent offenders (*n* = 19) Non-offender controls (*n* = 24)	31.4 ± 8.7 32.4 ± 10.7	100% ♂	8.9 ± 2.5 11.3 ± 1.1 education	PET	ROI: anterior cingulate cortex, OFC, and striatum Controlling for age and IQ		In the violent offender group, striatal 5-HT1BR binding was positively correlated with trait anger (non-reported statistics)
Hofhansel et al., 2020	Violent offenders (*n* = 27) Non-offender controls (*n* = 27)	35.59 ± 9.47 34.37 ± 10.30	100% ♂	10.44 ± 0.93 14.15 ± 2.40	MRI (gray matter volume), 3 T	- ROI: whole brain analysis Controlling total intracranial volume		Reactive or proactive aggression was unrelated to PFC GMV in offenders or controls (statistics non-provided)
Hoptman et al., 2005	SZ with LHA (e.g., violent attacks against others) (*n* = 46)	41.5 ± 8.2	96% ♂	-	MRI (gray and white matter volume), 1.5 T	ROI: OFC -		Total aggression severity score was positively associated with GMV of the left OFC (*r* =.48, *p* =.007) and larger left and right OFC WMV (*p* < 0.02). Finally, hostility subscale positively associated with larger leftward asymmetry in OFC WMV (*p* =.04)
Leutgeb et al., 2015	Violent offenders (*n* = 40) Non-offender controls (*n* = 37)	38.1 ± 12.0 36.7 ± 9.6	100% ♂	11.4 ± 1.9 11.6 ± 1.4	MRI (gray matter volume), 3 T	ROI: DLPFC, DMPFC, OFC amygdala, insula, caudate nucleus, pallidum, putamen, cerebellar hemispheres, vermis, and SMA -		In prisoners, GMV of left and right OFC positively correlated with violence recidivism (non-reported statistics) In controls, STAXI Anger Control negatively correlated with GMV of left OFC (non-reported statistics)
**Functional**								
**Life history of aggression**								
**Authors**	**Sample**	**Age**	**Gender**	**Educational level and handedness**	**Neuroimaging characteristics**	**Laboratory task**	**ROI and covariates**	**Results**
McCloskey et al., 2016	IED with LHA (e.g., violent attacks against others) (*n* = 20) Healthy controls (*n* = 20)	33.2 years 32.8 years	60% ♂ 60% ♂ 100% Right-handed	15.0 ± 1.7 15.9 ± 1.9	fMRI, 3 T	Angry (or Happy) face vs. Neutral face (emotional faces)	ROI: amygdala and VMPFC/ OFC -	Absence of significant correlation between PFC regions with LHA
Goyer et al., 1994	Personality disorders with LHA (e.g., Aggressive behavior directed toward others) (*n* = 17)	From 18 to 31	71% ♂	-	PET (metabolic rates of glucose)	Resting	ROI: frontal cortex -	Cerebral metabolic rates of glucose of OFC associated with LHA (*r* = -.54, *p* <.03) Cerebral metabolic rates of glucose of anterior medial frontal cortex (part of DMPFC and DLPFC) associated with LHA (*r* = -.63, *p* <.01)
**Aggression**								
**Trait aggression and anger expression (e.g., reactive, proactive or out)**								
**Resting**								
Gan et al., 2019 (overlapped with Gan et al., 2016)	IED with high anger trait (*n* = 11) Healthy controls (*n* = 12)	35.36 ± 7.4 30.7 ± 5.2	100% ♂ 100% ♂	13.5 ± 1.7 13.9 ± 1.4	fMRI, 4 T	rsFC	ROI: whole brain analysis -	IED group, activation of the left DLPFC with trait aggression (*r* = −.039, *p* =.909) Controls, activation of left DLPFC with trait aggression (*r* = −.294, *p* =.353)
Kolla et al., 2021	ASPD offenders (*n* = 16) Healthy controls (*n* = 16)	35.6 ± 9.4 30.0 ± 9.5	100% ♂ 31% with SZ with each group	13.0 ± 2.3 16.0 ± 2.2	PET, 3 T	Resting	ROI: whole brain analysis -	Absence of significant correlations between fatty acid amide hydrolase in OFC with trait and reactive aggression (statistics non provided)
Lanctôt et al., 2004	Violent patients with Alzheimer (*n* = 30) Non-violent patients with Alzheimer (*n* = 19)	74.0 ± 10.7	51% ♂	-	SPECT	Resting	ROI: OFC, middle and inferior regions of medial temporal gyrus, anterior cingulate corte and hypothalamus) -	Correlation in aggressive group between right rCBF in OFC with anger expression out (*n* = 30; *r* = -.42, *p* =.02)
Wolfs et al., 2023	Violent veterans (*n* = 19) Non-violent veterans (*n* = 22)	35.0 29.5	100% ♂	-	fMRI, 3 T	rsFC	ROI: whole-brain analysis -	For all the sample (*n* = 41), functional connectivity between deep cerebellar nuclei with left OFC was negatively correlated with trait aggression (*r* = −0.32, *p* = 0.043), but unrelated to trait aggression measured with STAXI (*r* = −0.17, *p* =.274)
**Laboratory task**								
Bertsch et al., 2018	BPD with anger expression tendency (*n* = 30) Healthy controls (*n* = 28)	26.9 ± 6.1 26.5 ± 5.7	100% ♀	-	fMRI, 3 T	Approach-avoidance task (faces)	ROI: left and right lateral anterior PFC (aPFC) and DLPFC and amygdala´ -	In borderline patients, aPFC activation and decreased in the right amygdala were unrelated to anger expression-out. In healthy volunteers, congruency effects decreased in the left aPFC and increased in the right amygdala with anger expression-out
Bertsch et al., 2019	BPD with anger expression tendency (*n* = 15) Healthy controls (*n* = 25)	28.3 ± 8.9 30.1 ± 5.9	100% ♂	-	fMRI, 3 T	Approach-avoidance task (faces)	ROI: left and right aPFC and DLPFC. Coupling of left and right aPFC and DLPFC with the amygdala -	The congruency effect in these three prefrontal areas was negatively correlated with anger expression-out in the patient group (right aPFC: *r* = −.63; left dlPFC: *r* = −.38, right dlPFC: *r* = −.68), while no negative correlation was found in the healthy control group (right aPFC: *r* =.09; left DLPFC: *r* =.39, right DLPFC: *r* =.19) Neither the connectivity analyses with the left and right aPFC as seed regions nor additional connectivity analyses with the left and right DLPFC as seeds revealed any significant coupling with the amygdala
Herpertz et al., 2017	BPD men with anger expression tendency (*n* = 23) BPD female with anger expression tendency (*n* = 33) Non-forensic control men (*n* = 26) Non-forensic control female (30)	30.65 ± 8.75 26.19 ± 5.70 31.09 ± 6.65 27.69 ± 6.38	100% ♂ - 100% ♂ -	10.87 ± 1.36 11.64 ± 1.46 11.72 ± 1.43 12.21 ± 1.19	fMRI	Script-driven-imagery paradigm (audio)	ROI: amygdala, ACC, OFC, and DLPFC -	In female patients, the coupling between left amygdala and the right DLPFC positively correlated with trait anger In male patients, the coupling between left amygdala with the right ventrolateral PFC and left OFC negatively correlated with trait anger
Smaragdi et al., 2019 (overlapped with Kolla et al., 2015)	BPD with high anger trait (*n* = 16) ASPD with high anger trait (*n* = 15) Healthy controls (*n* = 21)	37 ± 10.3 35 ± 9.1 34 ± 7.7	56% ♂ 100% ♂ 100% ♂	-	fMRI, 3 T	-	ROI: ACC and DLPFC -	Relationship between glutamine levels of the DLPFC with anger expression levels in ASPD (*r* =.59, *p* =.026) Necessary considering MAO-A We found significantly elevated levels of glutamine in the ASPD group relative to the BDP and healthy control groups in the DLPFC (*p* =.014), and a positive correlation between Glx levels and aggression in the DLPFC in the ASPD group alone
Fortier et al., 2023	SZ (*n* = 44) Healthy controls (*n* = 22)	34.30 ± 1.44 31.86 ± 1.79	100% ♂	92% right-handed 95% right-handed	fMRI, 3 T	Emotional Go/NoGo task (actors portraying anger (Angry) or no emotion (Neutral)	ROI: DLPFC, MFG, OFC, dACC and anterior insula -	In SZ, left MFG and right lateral OFC unrelated with violent behavior (Wald χ^2^ = 0.96 *p* = 0.39). In controls, left MFG and right lateral OFC unrelated with aggressive behavior (Wald χ^2^ = 2.47 *p* = 0.23)
Moeller et al., 2014	IED (*n* = 11) Cocaine (*n* = 21) Healthy controls (*n* = 17)	33.5 ± 7.1 43.2 ± 6.5 32.6 ± 6.4	100% ♂ 100% Right-handed	13.5 ± 1.7 13.0 ± 2.0 13.5 ± 1.4	fMRI, 4 T	Color-word Stroop task	ROI: whole brain analysis -	Across all participants, there was a positive association between the anterior DLPFC activation to errors with anger expression-out (*r* =.41, *p* =.004)
Tonnaer et al., 2017	Violent offenders (*n* = 16) Non-offender controls (*n* = 18)	35.81 ± 7.17 34.39 ± 13.37	100% ♂	89% secondary 88% secondary	fMRI, 3 T	Anger provocation and regulation task	ROI: whole brain analysis -	For all the sample, (*n* = 34), less activity in the VLPFC during anger regulation is associated with total anger expression; (*r* = −.47, *p* =.005) for reactive anger expression (*r* = −.44, *p* *=*.01) and for proactive anger expression (*r* = -.44, *p* =.01) and for trait anger (*r* = −.45, *p* =.005)

*Note*: *ACC* anterior cingulate cortex, *ASPD* antisocial personality disorder, *BDP* borderline personality disorder, *dACC* dorsal anterior cingulate cortex, *DLPFC* dorsolateral prefrontal cortex, *DMPFC* dorsomedial prefrontal cortex, *DZ* dizygotic, *FDG* F-fluorodeoxyglucose, *fMRI* functional magnetic resonance imaging, *GMD* gray matter density, *GMV* gray matter volume, *IED* intermittent explosive disorder, *IQ* intelligent quotient, *LHA* life history of aggression, *MFG* middle frontal gyrus, *MRI* magnetic resonance imaging, *MTG* middle temporal gyrus, *MZ* monozygotic, *OFC* orbitofrontal cortex, *PFC* prefrontal cortex, *PET* positron emission tomography, *rsFC* resting state functional connectivity, *SZ* schizophrenia, *SMA* supplementary motor area, *PFC* prefrontal cortex, *VLPFC* ventrolateral prefrontal cortex, *VMPFC* ventromedial prefrontal cortex, *WMV* white matter volume

First, the analysis of life history of aggression, mainly measured with the Life History of Aggression Scale-Revised (Coccaro et al., 1997), specifically focused on the subscale of aggression describing violent attacks against others, allowed us to conclude that certain regions of the PFC seemed to be partly involved in its explanation, as indicated by a relatively low correlation coefficient and the percentage of explained variance, maintaining both an inverse association. However, it is important to specify and highlight which regions contribute to it. Concretely, several studies signal the contribution of the OFC to life history of aggression. Accordingly, in borderline patients, GMV of the OFC (bilaterally) correlated negatively with this variable (Drachman et al., 2022), but the combination of several psychiatric disorders (e.g., schizophrenia, schizoaffective, anxiety, alcohol use disorder, among others) allowed us to conclude that only the GMV of the left OFC inversely correlated with life history of aggression (Gansler et al., 2009). Nonetheless, GMV in the medial and lateral PFC negatively correlated with life history of violence in violent individuals without mental disorders (Coccaro et al., 2018). These conclusions were complemented by another study which measured the cortical thickness in violent individuals. The study concluded that the superior frontal gyrus (or the superior part of the PFC) was negatively associated with life history of aggression (Bounoua et al., 2022).

There were also several studies assessing whether PFC structure explained trait aggression or externalization of anger (anger expression-out). In fact, four studies were included in this section, one of them also included in the above-described section. From these, only two failed to report significant associations between those variables in psychiatric patients and violent offenders (Antonucci et al., 2006; Hofhansel et al., 2020) and the other two that reported differences were relatively congruent. Both concluded that the GMV of the left OFC was positively associated with the risk of violence recidivism in patients with schizophrenia (Hoptman et al., 2005) and in violent offenders (Leutgeb et al., 2015). However, certain discrepancies existed between both. While Hoptman et al., (2005) concluded that only the left hemisphere was related to violence recidivism, Leutgeb et al., (2015) asserted that both hemispheres were significantly and positively associated with violence proneness. Last, the white matter volume of the OFC (bilaterally) entailed higher violence proneness in patients with schizophrenia (Hoptman et al., 2005).

From the studies measuring PFC activation during a resting period or in response to laboratory tasks and their association with trait aggression, a great part of the studies focused on BPD patients (Bertsch et al., 2018; Bertsch et al., 2019; Herpertz et al., 2017; Smaragdi et al., 2019). Compared to previous sections, there was a clear distinction between men and women or, at least, the effect of gender was controlled. It gave the impression that the activation of the right anterior PFC and the DLPFC (bilaterally) in an emotional laboratory task only presented an inverse association with anger expression-out in men with BPD (Bertsch et al., 2019). Nonetheless, the activation of the anterior DLPFC in an inhibitory control task was positively associated with anger expression-out in men with IED, misusers of cocaine and controls (Moeller et al., 2014). Continuing with IED patients, the lower activation of the VLPFC during stressful tasks was associated with higher levels of life history of aggression (Gorka et al., 2018), as well as with higher anger expression and trait levels in violent and control men (Tonnaer et al., 2017). Furthermore, a reduced cerebral metabolic rate of glucose in the OFC entailed higher levels of life history of aggression in patients with IED (Goyer et al., 1994). Finally, an additional study failed to report a significant association between glutamine levels of the DLPFC and anger expression levels in men and women with BPD (Smaragdi et al., 2019). In fact, the glutamine levels of the DLPFC only correlated positively and significantly with anger expression in men with ASPD (Smaragdi et al., 2019).

Regarding functional connectivity, two studies failed to report a significant association between the anterior PFC and the amygdala in men (Bertsch et al., 2018) or women with BPD (Bertsch et al., 2019), especially when assessing the connectivity of those brain regions with an approach-avoidance task related to facial emotional stimuli. Conversely, in a study that employed an emotional induction based on a script-driven task revealed that while the coupling between the left amygdala and the right VLPFC and lateral OFC negatively correlated with trait anger in male patients, in female patients, the coupling between the left amygdala and the right DLPFC positively correlated with trait anger (Herpertz et al., 2017). This was also corroborated by an additional study with violent patients with schizophrenia, concluding that a reduced connectivity between the amygdala and the VLPFC (bilaterally) entailed higher levels of life history of aggression as well as number of arrests in those patients (Hoptman et al., 2010). At the end, the reduced rsFC between the deep cerebellar nuclei and the left OFC was negatively correlated with trait aggression in violent veterans (Wolfs et al., 2023) (Table 4).

### Meta-analytic and complementary approach of the association between PFC subregions with different forms of violence against others

#### OFC

With regard to the association between OFC and different forms of violence, there was an inverse and non-significant association with violence in a sample of 149 participants with mental and personality disorders comprising 6 studies (*r* = -.29, 95% CIs [-.66,.19]; *Z* = −1.57, *p* =.058), with results across studies being heterogeneous (Tau^2^ =.31; *Q* = 35.25, *p* <.001; I^2^ = 85.81%) (Supplementary Table 2). The analysis of publication bias based on the Egger regression revealed the following values (Egger’s test = -.54, *p* =.62; Begg & Mazumdar, *Z* =.19, *p* =.851). After removing the study considered as an outlier (Hoptman et al., 2005), the relationship between variables was inverse and significant (*r* = -.51, 95% CIs [-.63, -.37]; *Z* = −8.92, *p* <.001; with results across studies being homogeneous (Tau^2^ =.00; *Q* = 1.39, *p* =.846; I^2^ = 0%; and without publication biases as revealed by Egger regression (Egger’s test = 1.47, *p* =.24; Begg & Mazumdar, *Z* =.49, *p* =.624).

#### DLPFC

The analysis between both variables allowed us to conclude the existence of an inverse and significant association with violence in a sample of 320 participants with and without mental and personality disorders comprising 4 studies (*r* = -.27, 95% CIs [-.38, -.16]; *Z* = −5.57, *p* <.001), with results across studies being homogeneous (Tau^2^ =.00; *Q* = 5.21, *p* =.634; I^2^ = 0%). (Supplementary Table 3). This association did not present publication biases following the Egger regression analysis (Egger’s test = -.14, *p* =.89; Begg & Mazumdar, *Z* =.74, *p* =.458).

#### DMPFC

With regard to the DMPFC and its association with violence, the existence of an inverse and significant association with violence in a sample of 122 participants with and without mental and personality disorders could be concluded comprising 4 studies (*r* = -.49, 95% CIs [-.59, -.36]; *Z* = −8.77, *p* <.001), with results across studies being homogeneous (Tau^2^ =.00; *Q *= 1.90, *p* =.863; I^2^ = 0%) (Supplementary Table 4). This association did not present publication biases according to the Egger regression analysis (Egger’s test = 1.56, *p* =.19; Begg & Mazumdar, *Z* =.56, *p* =.573).

## Discussion

Based on the empirical studies included in this systematic review, we collected a total of 86 empirical articles (some of which overlapped) to present the following conclusions. The results allowed us to conclude that there were differences in the reduced GMV in the OFC (bilaterally). Specifically, this was observed in certain violent groups, such as murderers, offenders without mental disorders who committed violent crimes, patients with intermittent explosive disorder (IED), antisocial personality disorder (ASPD) and psychopaths who perpetrated serious violent attacks against others, when compared to controls (healthy non-offenders). Additionally, gyrification abnormalities were also found in the OFC of patients with schizophrenia who perpetrated serious violent attacks against others. Other structural differences noted in different studies were lower GMV in the medial (DMPFC and VMPFC) and lateral PFC (DLPFC) among violent offenders without mental disorders, individuals with ASPD, and psychopaths. During resting periods, only murderers and patients with IED exhibited a reduced glucose metabolism in PFC bilaterally. Furthermore, there are other alterations that affect the 5-HT neurotransmission in PFC. Accordingly, violent offenders without mental disorders exhibited lower synthesis in the OFC bilaterally, while patients with IED exhibited higher levels of 5-HT2A receptors in this PFC region, and so did violent offenders in the DLPFC. Several generalizable patterns of resting state functional connectivity (rsFC) were related to a proneness to violence. Specifically, connections between the OFC and the amygdala and cerebellum, as well as between the lateral PFC (DLPFC and VLPFC) and the amygdala, were identified. Additionally, alterations in emotional processing were associated with a reduced activation of the OFC and VMPFC in violent offenders without mental disorders, violent schizophrenia patients, individuals affected by IED and depressive violent patients. Furthermore, anger engagement was associated with higher DLPFC activation in violent offenders, IED and BPD patients, as well as psychopaths. Finally, the complementary meta-analytic approach allowed us to conclude a significant inverse association between volume and blood flow in the OFC, DLPFC, and DMPFC with different forms of violence, especially a history of aggression.

The analysis of the risk of bias from the included empirical research based on nine criteria allowed us to conclude that many of these studies exhibited a moderate risk of bias, approximately 68% of the studies, followed by 22% of the studies having a low risk of bias and, finally, 10% of the research having a high risk of bias (see Supplementary Table 1). In this sense, it could be highlighted that much of the research did not justify the employment of a reduced sample size, providing few variables describing the participants (e.g., sociodemographic variables). In fact, only a few studies employed a sample size equal to or higher than 50 participants per group. Additionally, many of them neglected to describe the time frame for conducting their study (number of sessions, separation between sessions, among others). Nevertheless, all these studies make an important effort to analyze at-risk populations by using reliable instruments. Therefore, it is essential to carefully consider these limitations to improve future research and overcome current limitations.

Regarding the main objectives of this systematic review, we initially planned to summarize the main outcomes of neuroimaging research studies measuring the morphology and/or functioning of the PFC (total or subregions) in different samples of violent and/or criminal adults compared to non-violent individuals (or controls). We also tried to examine the potential association between the PFC (morphology and/or functioning) and violent behavior (or related concepts to this construct) in adults. This would allow us to conclude which PFC subregions, and their connections, might characterize the different samples of violent individuals, as well as those specific to each violent group. Accordingly, we were able to conclude that some PFC alterations could be generalized to different subsamples of individuals with and without mental or personality disorders who perpetrated serious violent attacks against others. First, it seemed that reduced GMV in the OFC, often bilaterally, might apply to different samples of violent individuals. In fact, these conclusions were supported by comparative studies and those using correlation or regression analysis. Moreover, violent patients with schizophrenia showed higher levels of gyrification in this region, which has been interpreted as a deviation in the neurodevelopmental process (Sasabayashi et al., 2020). The role of this subregion of the PFC in violence proneness may be explained by its critical involvement in executive functioning and the maintenance of hostile cognitive schemas (Romero-Martínez et al., 2024b). Additionally, patients with OFC lesions often have significant difficulties recognizing emotional expressions (Jonker et al., 2015), along with behavioral disinhibition and impulsivity (Blake & Grafman, 2010). This aligns with several studies included in this systematic review, which indicated that the OFC showed diminished activation when responding to emotional stimuli (Alia-Klein et al., 2014; Coccaro et al., 2007; Fortier et al., 2023; Joyal et al., 2007). All these processes, combined with hostile cognitive schemas, may facilitate emotional and impulsive violence. However, it should be noted that this cannot be established from a localizationist perspective without considering other factors or variables affecting the OFC, as well as other PFC regions and their interconnections.

Sustained OFC alterations and its association with impulsive violence, two groups of individuals prone to impulsive violent aggression were identified: those diagnosed with IED of both genders (Booij et al., 2010; Rosell et al., 2010), as well as impulsive and violent individuals of both genders who presented a reduced number of 5-HT2A receptors in the DLPFC (Meyer et al., 2008). Abnormalities in 5-HT synthesis and the number of postsynaptic receptors (upregulated) are, therefore, considered a strong cause or underlying factor of mood dysregulation (Eison and Mullins, 1995). Regarding the volumetric association of OFC with violence intake, the meta-analytic approach based on approximately 100 participants, after removing the study considered an outlier, revealed that reduced volume in the OFC was associated with high violence intake. In fact, the correlation coefficient could be considered as high.

To adequately understand why these individuals may be prone to violence due to alterations in the OFC (morphological and 5-HT functioning), it is also important to consider their functional connectivity with other brain structures. Several studies have shown that violent individuals often have an inverse relationship between the OFC and the cerebellum and amygdala (Coccaro et al., 2007; Leutgeb et al., 2016; Wolfs et al., 2023), although there are inconsistencies regarding which hemisphere shows this relationship. This inverse association between the prefrontal cortex (PFC) and other brain structures has been observed not only at rest (Leutgeb et al., 2016; Wolfs et al., 2023) but also during emotional processing (Coccaro et al., 2007). These changes may reflect the significant challenges some violent individuals with a history of violence face in integrating, coping with, and responding to emotional stimuli. Additionally, some violent individuals show reduced OFC activation when dealing with emotional stimuli or when they need to inhibit certain responses (Alia-Klein et al., 2014; Coccaro et al., 2007; Joyal et al., 2007; Lee et al., 2008). This reduced OFC activity is accompanied by increased activity in areas such as the cerebellum (Wolfs, 2023) and amygdala (da Cunha-Bang et al., 2017a) when processing emotional stimuli or during episodes of anger. This may indicate the difficulties violent individuals experience when driven by emotional stimuli and trying to suppress undesirable responses.

Regarding the lateral and medial PFC, there was less consistent evidence across groups. However, some conclusions could be drawn. For example, it could be stated that violent offenders without mental disorders, those with IED, and psychopaths, tend to present reduced GMV in the DMPFC (Bertsch et al., 2013; Coccaro et al., 2016, 2018; Leutgeb et al., 2015; Seok & Cheong, 2020), whereas low GMV in the DLPFC could also be attributed to violent offenders with ASPD as well as those with ASPD and BPD (Bertsch et al., 2013; Leutgeb et al., 2016; Raine et al., 2011). The meta-analysis of both subregions of the PFC revealed a significant inverse association between volumetric measurements and violence facilitation. While this conclusion was homogeneous in both cases, the correlation coefficient was low for the DLPFC and moderate for the DMPFC. Additionally, the conclusion regarding the DLPFC was based on a larger sample size compared to the DMPFC. Interestingly, DMPFC contains the ‘dorsal nexus’, which constitutes a key intersection for multiple brain networks (Sheline et al., 2010). In fact, the abnormal activation of this region results in a heightened tendency to ruminative processes of different content (e.g., including anger or revenge thoughts) (Kim et al., 2023; Peters et al., 2018), that in turn, tend to facilitate violence proneness, especially, after being provoked (Pedersen et al., 2011). Not only structural differences have been found, but also functional. Violent offenders exhibited a diminished DMPFC activation when processing emotional stimuli (Pardini & Phillips, 2010), as well as reduced rsFC between this PFC region and the middle temporal gyrus and the left amygdala when faced with a frustrating task (Hofhansel et al., 2023; Siep et al., 2019). Furthermore, under resting conditions, violent individuals also exhibited a diminished rsFC between the left DMPFC and left precuneus and left occipital regions (Gan et al., 2019). These differences in DMPFC activation combined with variations in connectivity patterns with other brain regions and structures related to information integration might verify serious difficulties integrating and interpretating stimuli, which tend to increase and reaffirm the self-ruminative process.

The other PFC region, DLPFC, also presented lower GMV in violent offenders (Leutgeb et al., 2016), those with ASPD (Raine et al., 2011), as well as ASPD offenders with BPD (Bertsch et al., 2013). Together with the VLPFC and other brain structures, this PFC region is involved in cognitive reappraisal (Etkin et al., 2015). A study concluded that violent offenders showed greater activity in the left DLPFC and VLPFC during anger engagement, suggesting that they reinterpret environmental stimuli in a hostile way, which facilitates their anger (Tonnaer et al., 2017). Furthermore, violent patients with schizophrenia and those with IED also exhibited a diminished activation of the right DLPFC during an inhibiting task with emotional content (Tikàsz et al., 2018). Taken together, this differential activation when coping with anger or inhibiting inappropriate responses compared to controls, which entails a heightened risk for violence, was reinforced by the inverse association of the DLPFC with limbic structures such as the basolateral amygdala in violent offenders (Varkevisser et al., 2017) or a heightened connectivity during resting periods in the dorsal striatum (Amaoui et al., 2022), a key structure for decision-making processes, specifically, action selection and initiation (Balleine et al., 2007). Furthermore, it seemed that the DLPFC maintains a heightened connection bilaterally in violent individuals (Leutgeb et al., 2016). Hence, it could be established that violent offenders tend to make mistakes when selecting targets or responses to cope with the environment and, as a result, struggle to inhibit or avoid inappropriate responses such as violent reactions.

With regard to the VLPFC, it has been established that this PFC region represents an important contribution to threat detection, especially through its connection with the amygdala (Monk et al., 2006; Nomura et al., 2004). Accordingly, the included research concluded that violent offenders, and violent patients with schizophrenia and psychopathy exhibited a heightened activation of the VLPFC during anger engagement and emotional processing (Müller et al., 2003; Schiffer et al., 2017; Tonnaer et al., 2017), except in response to stressful events, with a diminished VLPFC activation entailing a heightened risk for violent behavior in IED patients (Gorka et al., 2018). The heightened activation of the VLPFC during emotional engagement together with the analysis of rsFC, revealed that the left VLPFC tends to maintain a positive connectivity with the brainstem, middle temporal area and hippocampus, while the right VLPFC maintains an inverse association with the sensorimotor, premotor, intraparietal and occipital areas in violent offenders (Amaoui et al., 2022), as well as with the amygdala in patients with schizophrenia and men with BPD who have committed violent crimes (Herpertz et al., 2017; Hoptman et al., 2009). In this sense, the inverse coupling between the VLPFC and the amygdala entailed higher scores in anger trait, being this association especially relevant in men (Herpertz et al., 2017; Hoptman et al., 2009). All these results reinforce the hypothesis that the inverse connection between the VLPFC and limbic structures, such as the amygdala, might lead to misunderstandings of emotional stimuli with a hostile or threatening interpretation, potentially resulting in violent reactions, as stated above (Monk et al., 2006; Nomura et al., 2004).

Veterans with damage to the VMPFC exhibited higher proneness to violence (Grafman et al., 1996). The research included in our review did not support the view of major structural differences in the GMV of the VMPFC in violent individuals, except for BPD patients with ASPD (Bertsch et al., 2013), although empirical studies supporting the contribution of this PFC region existed. In this sense, it seemed that the diminished activation of the VMPFC was involved in anger engagement (Chester & DeWall, 2019; Dougherty et al., 2004; Gan et al., 2016). Due to the role of the VMPFC in regulating negative emotions (Hiser & Koenigs, 2018), it seems logical to conclude that the tendency toward negative affect might diminish the threshold for reacting with violence under uncomfortable circumstances.

Despite the interest of this systematic review focusing on adults, the different limitations that affected the review, along with those inherent in the included empirical research, should be analyzed in detail. It is important to note that considering a wide range of research with varying methodologies (e.g., neuroimaging techniques, ROI-based analyses, statistical control for multiple comparisons, potential differences between samples) increases the risk of biases in interpreting results. This, in turn, may lead to an overestimation of the central role of the PFC. Nevertheless, we conducted a complementary meta-analytic approach for the second aim of this review, and the conclusions aligned with those stated in the systematic review. This reinforces the view of the varying importance of each subregion of the PFC in relation to violence proneness. Another limitation of this systematic review might be attributed to the criteria established for conducting it. In other words, the employment of certain restrictive inclusion criteria that only allowed the employment of empirical research published in peer-reviewed journals, neglecting grey literature. Furthermore, we also avoided the analysis of studies based on normative individuals without life history of aggression or some form of tendency to express the anger, which would increase the number of studies included, but diminish their applicability for violent individuals biasing conclusions. Another limitation is the absence of studies measuring the structural connectivity, which might impact the interpretation of the results. Additionally, much of the included empirical research had a relatively small sample size (n ≤ 50 participants per group), mainly focused on men. Due to the relatively low number of studies publishing descriptive data, we were unable to calculate the effect size of the group differences or the potential association between variables. Therefore, it is important to consider this for future research to facilitate the calculation of these differences or potential associations. Moreover, we have detected a considerable number of studies which present an overlapping of data, so we removed a considerable number of studies to avoid biasing conclusions. Another limitation affects the employment of a whole brain analysis with a reduced sample size. The conclusions highlighted in this review might help guide future research and establish specific ROIs for conducting this kind of studies with violent individuals. Lastly, another limitation of the included research is the absence of longitudinal studies that establish whether the functional connectivity or response of these individuals to different types of laboratory tasks persist over time or are specific to a particular moment.

In conclusion, this systematic review found that some subregions of the PFC might be characteristic of different groups of violent individuals together with other specific ones of each sample. For example, much of the research concluded that there is a reduced volume of the OFC in some samples of violent individuals with and without mental or personality disorders, showing a clear association with violence proneness. This structural alteration in the OFC is associated with 5-HT neurotransmission alterations. Not only were structural differences observed, but also other functional connectivity during resting periods, which involved dysfunctions in the connectivity between the PFC and the amygdala and cerebellum. It could also be concluded that violent individuals tend to show reduced activation of the OFC and VMPFC during emotional processing, while exhibiting increased activation of the DLPFC when engaging in violence. All these conclusions, combined with those specific to the violent groups included in this research, might help build forensic profiles by integrating traditional methods and neuroimaging techniques. This, in turn, could focus on the PFC as a potential target for future research aimed at developing treatments to reduce violence in these individuals.

## Supplementary Information

Below is the link to the electronic supplementary material.

Supplementary file1 (DOCX 60 kb)

## Data Availability

No datasets were generated or analysed during the current study.
