# Supplementary material for: The analysis of the prefrontal cortex and its facilitator role of violence: conclusions of a systematic review and meta-analysis based on neuroimaging results
Source: Brain Imaging Behav. 2026 Mar 10;20(2):41. doi: 10.1007/s11682-026-01105-1 (PMC12975852; doi:10.1007/s11682-026-01105-1)
Supplement: Supplementary file 1 — Supplementary file1 (DOCX 60 kb) [file 11682_2026_1105_MOESM1_ESM.docx]

|  | |  | | **Supplementary table 1.**  Summary of design quality for included studies alphabetically ordered and by the year of publication | | | | | | | | | | |
| --- | --- | --- | --- | --- | --- | --- | --- | --- | --- | --- | --- | --- | --- | --- |
|  | |  | |  | | | | | | | | | | |
| **Authors** | | | **Criterion** | | | | | | | |  | | **Risk of bias** | |
|  |  |  | **Objectives and hypothesis** | | **Sample** | | | **Procedure** | **Results** | | | | |  |
|  |  |  | 1a | | 2a | 2b | 2c | 3a | 4a | 4b | 4c | 4d | |  |
| Alia-Kelin et al., 2014 | | | No | | No | No | Yes | Yes | Yes | - | Yes | No | | Moderate |
| Amaiou et al., 2022 | | | Yes | | Yes | No | Yes | Yes | No | Yes | Yes | Yes | | Low |
| Antonucci et al., 2006 | | | Yes | | No | No | Yes | No | No | No | No | No | | High |
| Barkataki et al., 2006 | | | Yes | | No | No | Yes | No | No | Yes | Yes | No | | Moderate |
| Basoglu et al., 2008 | | | Yes | | No | No | Yes | Yes | Yes | - | Yes | No | | Moderate |
| Bertsch et al., 2013 | | | No | | No | No | Yes | No | Yes | - | Yes | No | | Moderate |
| Bertsch et al., 2018 | | | No | | No | No | Yes | Yes | Yes | - | Yes | Yes | | Low |
| Bertsch et al., 2019 | | | Yes | | No | No | Yes | Yes | Yes | Yes | Yes | Yes | | Low |
| Birbaumer et al., 2005 | | | No | | No | No | Yes | No | Yes | No | Yes | No | | Moderate |
| Boccardi et al., 2011 | | | Yes | | No | No | Yes | No | No | No | Yes | Yes | | Moderate |
| Booij et al., 2010 | | | Yes | | No | No | Yes | Yes | Yes | Yes | No | No | | Moderate |
| Bounoua et al., 2022 | | | No | | No | Yes | Yes | Yes | No | Yes | Yes | Yes | | Low |
| Chang et al., 2017 | | | No | | No | No | Yes | No | No | No | Yes | No | | High |
| Chester y Dewall, 2019 | | Yes | | No | No | No | Yes | Yes | No | Yes | Yes | | Moderate |  |
| Coccaro et al., 2007 | | | Yes | | No | No | Yes | No | Yes | - | Yes | Yes | | Low |
| Coccaro et al., 2016 | | | No | | No | Yes | Yes | No | No | Yes | Yes | Yes | | Moderate |
| Coccaro et al., 2018 | | | No | | Yes | Yes | Yes | Yes | No | Yes | Yes | No | | Low |
| Critchley et al., 1999 | | | No | | No | No | No | No | No | Yes | Yes | No | | High |
| da Cunha-Bang et al., 2017a | | | Yes | | No | No | Yes | Yes | No | No | Yes | No | | Moderate |
| da Cunha-Bang et al., 2017b | | | Yes | | No | No | Yes | No | No | Yes | Yes | Yes | | Moderate |
| Dougherty et al., 2004 | | | Yes | | No | No | Yes | No | Yes | - | Yes | No | | Moderate |
| Drachman et al., 2022 | | | No | | No | No | Yes | No | Yes | No | No | No | | High |
| Fortier et al 2023 | | | No | | No | No | Yes | Yes | Yes | - | Yes | Yes | | Low |
| Gan et al., 2016 | | | Yes | | No | No | Yes | Yes | No | No | Yes | No | | Moderate |
| Gan et al., 2019 | | | Yes | | No | No | Yes | Yes | No | No | Yes | Yes | | Moderate |
| Gansler et al., 2009 | | | Yes | | No | No | Yes | Yes | No | No | Yes | No | | Moderate |
| George et al., 2004 | | | No | | No | No | Yes | No | No | No | Yes | No | | High |
| Geurts et al., 2016 | | | Yes | | No | No | Yes | Yes | Yes | - | No | No | | Moderate |
| Gorka et al., 2018 | | | No | | No | No | Yes | Yes | No | Yes | No | Yes | | Moderate |
| Goyer et al., 1994 | | | No | | No | No | Yes | Yes | No | Yes | Yes | No | | Moderate |
| Gregory et al., 2012 | | | Yes | | No | No | Yes | Yes | No | Yes | Yes | Yes | | Low |
| Gregory et al., 2015 | | | Yes | | No | No | Yes | Yes | No | No | Yes | No | | Moderate |
| Heesink et al., 2018 | | | Yes | | No | No | Yes | No | Yes | - | Yes | No | | Moderate |
| Herpertz et al., 2017 | | | Yes | | No | No | Yes | Yes | Yes | Yes | Yes | Yes | | Low |
| Hofhansel et al., 2020 | | | Yes | | No | No | Yes | Yes | No | Yes | Yes | No | | Moderate |
| Hofhansel et al., 2023 | | | Yes | | No | No | Yes | Yes | No | No | No | Yes | | Moderate |
| Hoptman et al., 2005 | | | Yes | | No | No | Yes | No | - | Yes | Yes | No | | Moderate |
| Hoptman et al., 2010 | | | No | | No | No | Yes | No | No | No | Yes | No | | High |
| Joyal et al., 2007 | | | No | | No | No | Yes | No | No | No | Yes | No | | High |
| Kiehl et al., 2004 | | | Yes | | No | No | Yes | No | Yes | No | Yes | No | | Moderate |
| Kolla et al., 2014 | | | No | | No | No | Yes | No | Yes | Yes | No | No | | Moderate |
| Kolla et al., 2015 | | | Yes | | No | No | Yes | No | No | Yes | Yes | No | | Moderate |
| Kolla et al., 2017 | | | Yes | | No | No | Yes | No | Yes | - | No | Yes | | Moderate |
| Kolla et al., 2021 | | | Yes | | No | No | Yes | No | No | Yes | No | Yes | | Moderate |
| Kumari et al., 2006 | | | No | | No | No | Yes | No | No | Yes | Yes | Yes | | Moderate |
| Laakso et al., 2002 | | | No | | No | No | Yes | No | No | Yes | No | No | | High |
| Lam et al., 2017 | | | Yes | | No | No | Yes | No | No | Yes | Yes | No | | Moderate |
| Lanctot et al., 2004 | | | Yes | | No | No | Yes | No | Yes | Yes | Yes | Yes | | Low |
| Lee et al., 2008 | | | Yes | | No | No | No | No | Yes | - | No | No | | Moderate |
| Lee et al., 2009 | | | No | | No | No | Yes | Yes | Yes | - | Yes | No | | Moderate |
| Leutgeb et al., 2015 | | | Yes | | No | No | Yes | Yes | Yes | - | Yes | No | | Low |
| Leutgeb et al., 2016 | | | Yes | | No | No | Yes | Yes | Yes | - | Yes | No | | Low |
| Marín-Morales et al., 2022 | | | Yes | | Yes | No | Yes | Yes | No | Yes | Yes | Yes | | Low |
| McCloskey et al., 2016 | | | Yes | | No | No | Yes | No | Yes | - | Yes | No | | Moderate |
| Meyer et al., 2007 | | | No | | No | No | Yes | No | Yes | Yes | Yes | No | | Moderate |
| Moeller et al., 2014 | | | Yes | | No | No | Yes | No | No | Yes | Yes | No | | Moderate |
| Müller et al., 2003 | | | No | | No | No | Yes | No | - | No | Yes | Yes | | Moderate |
| Narayan et al., 2007 | | | No | | No | No | Yes | No | Yes | Yes | Yes | No | | Moderate |
| Nummenmaa et al., 2021 | | | Yes | | No | No | Yes | Yes | Yes | Yes | No | Yes | | Low |
| Pardini & Phillips, 2010 | | | No | | No | No | Yes | Yes | No | No | Yes | No | | Moderate |
| Prehn et al., 2013 | | | No | | No | No | Yes | No | Yes | - | No | Yes | | Moderate |
| Raine et al., 1994 | | | Yes | | No | No | Yes | No | Yes | No | Yes | Yes | | Moderate |
| Raine et al., 1997 | | | Yes | | No | No | Yes | No | Yes | - | Yes | No | | Moderate |
| Raine et al., 2000 | | | No | | No | No | Yes | No | Yes | - | Yes | Yes | | Moderate |
| Raine et al., 2011 | | | No | | No | No | Yes | No | ? | Yes | Yes | No | | Moderate |
| Raine et al., 2012 | | | No | | No | No | Yes | No | Yes | Yes | No | No | | Moderate |
| Rosell et al., 2010 | | | Yes | | No | No | Yes | No | Yes | - | Yes | Yes | | Low |
| Schiffer et al., 2011 | | | Yes | | No | No | Yes | No | No | Yes | Yes | Yes | | Moderate |
| Schiffer et al., 2014 | | | Yes | | No | No | Yes | No | Yes | - | Yes | Yes | | Low |
| Schiffer et al., 2017 | | | No | | No | No | Yes | No | Yes | - | Yes | No | | Moderate |
| Schneider et al., 2000 | | | No | | No | No | Yes | Yes | Yes | - | No | No | | Moderate |
| Seok & Cheong, 2020 | | | No | | No | No | Yes | No | Yes | - | No | Yes | | Moderate |
| Siep et al., 2019 | | | Yes | | No | No | Yes | Yes | Yes | - | No | Yes | | Low |
| Smaragdi et al., 2019 | | | Yes | | No | No | Yes | No | No | No | Yes | No | | Moderate |
| Spoont et al., 2010 | | | No | | No | No | Yes | Yes | Yes | - | No | No | | Moderate |
| Storvestre et al., 2019 | | | No | | No | No | Yes | Yes | No | Yes | Yes | No | | Moderate |
| Tiihonen et al., 2008 | | | No | | No | No | Yes | No | No | No | Yes | Yes | | Moderate |
| Tikàsz et al., 2018 | | | No | | No | No | Yes | No | No | Yes | No | No | | High |
| Tonnaer et al., 2017 | | | Yes | | No | No | Yes | Yes | Yes | - | Yes | Yes | | Low |
| Varkevisser et al., 2017 | | | Yes | | No | No | Yes | No | Yes | - | Yes | No | | Moderate |
| Varkevisser et al., 2021 | | | No | | No | No | Yes | No | Yes | Yes | Yes | Yes | | Moderate |
| Volkow et al., 1995 | | | Yes | | No | No | Yes | No | ? | - | No | Yes | | Moderate |
| Volman et al., 2016 | | | Yes | | No | No | Yes | Yes | Yes | Yes | No | No | | Moderate |
| Wolfs et al., 2023 | | | Yes | | No | No | Yes | No | Yes | - | Yes | Yes | | Low |
| Wong et al., 1997 | | | Yes | | No | No | Yes | No | Yes | - | Yes | No | | Moderate |
| Yang et al., 2010 | | | No | | No | No | Yes | No | Yes | Yes | No | No | | Moderate |

| **Supplementary table 2**  *OFC associated with different forms of violence* | | | | | | | | | | |
| --- | --- | --- | --- | --- | --- | --- | --- | --- | --- | --- |
| **Study** | | **Type of sample** | | **Brain measurement** | **Violence assessment** | **Statistical values** | | | | |
|  |  |  |  |  |  | **Sample size** | **r** | **95% CI LL** | **95% CI UL** | **Weight** |
| Antonucci et al., 2006 | | Affective disorder with LHA | | GMV | LHA | 8 | -.29 | -.88 | .64 | 12.85% |
| Antonucci et al., 2006 | | Non-Affective disorder with LHA | | GMV | LHA | 7 | -.35 | -.92 | .70 | 11.70% |
| Gansler et al., 2009 | | Psychiatric patients with LHA | | GMV | LHA | 41 | -.59 | -.76 | -.34 | 19.55% |
| Goyer et al., 1994 | | Personality disorders with LHA | | CMRG | LHA | 17 | -.54 | -.82 | -.04 | 17.22% |
| Lanctôt et al., 2004 | | Violent patients with Alzheimer | | rCBF | Anger expression-out | 30 | -.42 | -.69 | -.05 | 18.94% |
| Hoptman et al., 2005 | | SZ patients with LHA | | GMV | Trait aggression | 46 | .48 | .21 | .68 | 19.73% |
|  | |  | |  |  | 149 | -.29 | -.66 | .19 | 100% |
|  |  | | Heterogeneity: Tau² = .31; Q = 35.25, p < .001; I² = 85.81%; test for overall effect: Z = -1.57, p = .058 | | | | | | | |

Note. CI: confidence interval; CMRG: cerebral metabolic rates of glucose; GMV: gray matter volume; LHA: life history of aggression; LL: lower limit; OFC: orbitofrontal cortex; rCBF: regional cerebral blood flow; SZ: schizophrenia; UL: upper limit.

| **Supplementary table 3**  *DLPFC associated with different forms of violence* | | | | | | | | | | |
| --- | --- | --- | --- | --- | --- | --- | --- | --- | --- | --- |
| **Study** | | **Type of sample** | | **Brain measurement** | **Violence assessment** | **Statistical values** | | | | |
|  |  |  |  |  |  | **Sample size** | **r** | **95% CI LL** | **95% CI UL** | **Weight** |
| Bounoua et al., 2022 | | Non-forensic controls with LHA | | CT | LHA | 134 | -.29 | -.44 | -.13 | 44.26% |
| Coccaro et al., 2018 | | Non-forensic controls with LHA | | GMV | LHA | 41 | -.07 | -.38 | .25 | 12.84% |
| Coccaro et al., 2018 | | Non-forensic controls with LHA | | GMV | LHA | 41 | -.29 | -.56 | .03 | 12.84% |
| Coccaro et al., 2018 | | Non-forensic controls with LHA | | GMV | LHA | 32 | -.29 | -.59 | .08 | 9.80% |
| Coccaro et al., 2018 | | Non-forensic controls with LHA | | GMV | LHA | 32 | -.25 | -.56 | .12 | 9.0% |
| Gan et al., 2019 | | IED with high anger trait | | Resting activation | Trait aggression | 11 | -.04 | -.68 | .63 | 2.70% |
| Gan et al., 2019 | | Non-forensic controls | | Resting activation | Trait aggression | 12 | -.29 | -.78 | .41 | 3.04% |
| Goyer et al., 1994 | | Violent patients with Alzheimer | | rCBF | Anger expression-out | 17 | -.63 | -.86 | -.17 | 4.73% |
|  | |  | |  |  | 320 | -.27 | -.38 | -.16 | 100% |
|  |  | | Heterogeneity: Tau² = .00; Q = 1.39, p = .846; I² = 0%; test for overall effect: Z = -8.92, p < .001 | | | | | | | |

Note. CI: confidence interval; CT: cortical thickness; DLPFC: dorsolateral prefrontal cortex; GMV: gray matter volume; IED: intermittent explosive disorder; LHA: life history of aggression; LL: lower limit; rCBF: regional cerebral blood flow; UL: upper limit.

| **Supplementary table 4**  *DMPFC associated with different forms of violence* | | | | | | | | | | |
| --- | --- | --- | --- | --- | --- | --- | --- | --- | --- | --- |
| **Study** | | **Type of sample** | | **Brain measurement** | **Violence assessment** | **Statistical values** | | | | |
|  |  |  |  |  |  | **Sample size** | **r** | **95% CI LL** | **95% CI UL** | **Weight** |
| Antonucci et al., 2006 | | Affective disorder with LHA | | GMV | LHA | 8 | -.29 | -.88 | .64 | 4.81% |
| Antonucci et al., 2006 | | Non-Affective disorder with LHA | | GMV | LHA | 7 | -.35 | -.92 | .70 | 3.85% |
| Gansler et al., 2009 | | Psychiatric patients with LHA | | GMV | LHA | 41 | -.59 | -.76 | -.34 | 36.54% |
| Gansler et al., 2009 | | Non-forensic controls | | GMV | LHA | 19 | -.35 | -.71 | .16 | 15.38% |
| Goyer et al., 1994 | | Personality disorders with LHA | | GMV | LHA | 17 | -.54 | -.82 | -.04 | 13.46% |
| Lanctôt et al., 2004 | | Violent patients with Alzheimer | | rCBF | Anger expression-out | 30 | -.42 | -.69 | -.05 | 25.96% |
|  | |  | |  |  | 122 | -.49 | -.59 | -.36 | 100% |
|  |  | | Heterogeneity: Tau² = .00; Q = 1.90, p = .863; I² = 0%; test for overall effect: Z = -8.77, p < .001 | | | | | | | |

Note. CI: confidence interval; DMPFC: dorsomedial prefrontal cortex; GMV: gray matter volume; LHA: life history of aggression; LL: lower limit; rCBF: regional cerebral blood flow; UL: upper limit.
